# Timing of organogenesis support basal position of turtles in the amniote tree of life

**DOI:** 10.1186/1471-2148-9-82

**Published:** 2009-04-23

**Authors:** Ingmar Werneburg, Marcelo R Sánchez-Villagra

**Affiliations:** 1 Paläontologisches Institut und Museum der Universität Zürich, Karl Schmid-Strasse 4, 8006 Zürich, Switzerland

## Abstract

**Background:**

The phylogenetic position of turtles is the most disputed aspect in the reconstruction of the land vertebrate tree of life. This controversy has arisen after many different kinds and revisions of investigations of molecular and morphological data. Three main hypotheses of living sister-groups of turtles have resulted from them: all reptiles, crocodiles + birds or squamates + tuatara. Although embryology has played a major role in morphological studies of vertebrate phylogeny, data on developmental timing have never been examined to explore and test the alternative phylogenetic hypotheses. We conducted a comprehensive study of published and new embryological data comprising 15 turtle and eight tetrapod species belonging to other taxa, integrating for the first time data on the side-necked turtle clade.

**Results:**

The timing of events in organogenesis of diverse character complexes in all body regions is not uniform across amniotes and can be analysed using a parsimony-based method. Changes in the relative timing of particular events diagnose many clades of amniotes and include a phylogenetic signal. A basal position of turtles to the living saurian clades is clearly supported by timing of organogenesis data.

**Conclusion:**

The clear signal of a basal position of turtles provided by heterochronic data implies significant convergence in either molecular, adult morphological or developmental timing characters, as only one of the alternative solutions to the phylogenetic conundrum can be right. The development of a standard reference series of embryological events in amniotes as presented here should enable future improvements and expansion of sampling and thus the examination of other hypotheses about phylogeny and patterns of the evolution of land vertebrate development.

## Background

**Figure 1:**
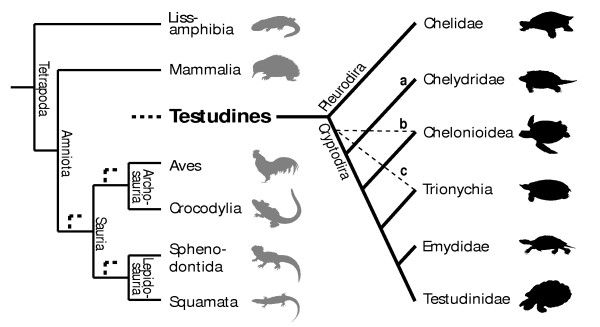
**Uncertain position of turtles in the amniote tree of life**. The three (out of eight) main hypotheses for the position of turtles within recent amniotes (bold dashed lines) and the alternative arrangements of cryptodire turtle groups compared in this study. a, b and c and light dashed lines indicate the most basal taxon according to each hypothesis. For all hypotheses compared see Figure S1 and S2 and Table S6.

The controversial phylogenetic position [1-5] of turtles among land vertebrates and their highly derived anatomical features [6-8] have been subject of molecular and morphological investigations. Three main hypotheses of sister-groups have resulted from them (Figure 1), if we ignore extinct taxa: the Sauria clade [9-11] or one of its subgroups, the Archosauria [4,12], with crocodiles and birds, or the Lepidosauria [13-15], with squamates and the tuatara. In view of this conflict, new lines of evidence to address this phylogenetic issue have been called for [16]. Although embryology has played a major role in morphological studies of vertebrate phylogeny and primary homology testing, data on developmental timing have never been examined to explore and test these conflicting phylogenetic hypotheses about turtle origin.

When examining the phylogenetic position of turtles among amniotes, relationships within turtles themselves are very relevant, as they determine the reconstruction of the last common ancestor of the clade. The subdivision of extant turtles into the side-necked pleurodires and the hidden-necked cryptodires is widely accepted, but no consensus exists on the systematics of the cryptodire subgroups. Alternative groupings of these clades are also included in our analysis (Figure 1a–c). Several additional hypotheses for the origin of turtles have been suggested in the past, but are no longer earnestly discussed, such as a close relationship of turtles to the remaining Amniota, to Aves, to Crocodylia, or to Mammalia [17].

**Figure 2:**
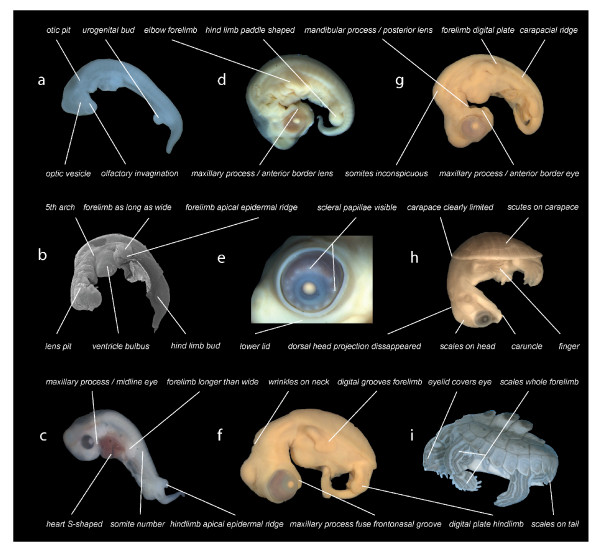
**Examples of developmental events examined**. Illustration of selected developmental events as used in this study. *Graptemys nigrinoda *(a, i), *Chelonia mydas *(b, f, g), *Emydura subglobosa *(c, h), *Lepidochelys olivacea *(d, e). Depicted elements refer to selected events as used in this study (see supplement). Embryos are of different ages and not to scale.

Following recent comparative embryological work [18] we defined 104 developmental characters of external morphology during organogenesis until hatching/birth based on a comprehensive review of the literature and our own study of embryological series (Figure 2). We examined 15 turtle and seven amniote species representing the other major extant amniote clades (see Additional file 1: Tables S1–S9, Figures S1–S5). One salamander was used as an outgroup. We included the first comprehensive data on side-necked turtle external embryology, represented by *Emydura subglobosa*. We implemented Parsimov [19], a parsimony-based method to quantify and compare sequence heterochrony, and used the number of timing shifts from the consensus data of the methods of character optimisation as a criterion to support the robustness of a particular branch [20-22]. There are 24 topologies on which Parsimov was implemented, resulting from eight alternative hypotheses for the position of turtles within Tetrapoda (Figure S1, Table S6), each combined with the three alternative topologies of the cryptodire subgroups (Figure 1a–c, Figure S2). In addition, a total of 5356 event pairs (= characters) were mapped: 3197 characters (59.7%) were constant, and 875 variable characters (16.3%) were parsimony-uninformative. The remaining 1284 characters (24%) were informative.

## Results and discussion

**Figure 3:**
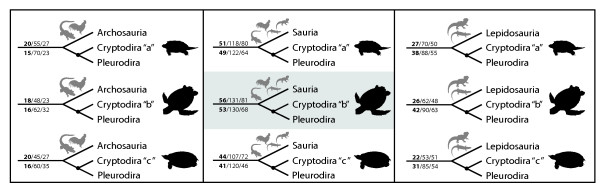
**Alternative hypotheses of turtle relationships tested**. The three (out of eight) main hypotheses on the position of turtles within Amniota combined to the three alternatives (a, b, c) of cryptodire relationships. Nodes indicate Testudines. The preferred hypothesis of our study is highlighted. Illustrations show the most basal cryptodire taxa in each hypothesis. The numbers above the main branch are the counts of CONSENSUS/ACCTRAN/DELTRAN shifts for the analysis with unordered characters, numbers below the main branch are the same counts for the analysis with ordered characters. For all hypotheses compared see Figure S1 and S2 and Table S6 and S7.

**Figure 4:**
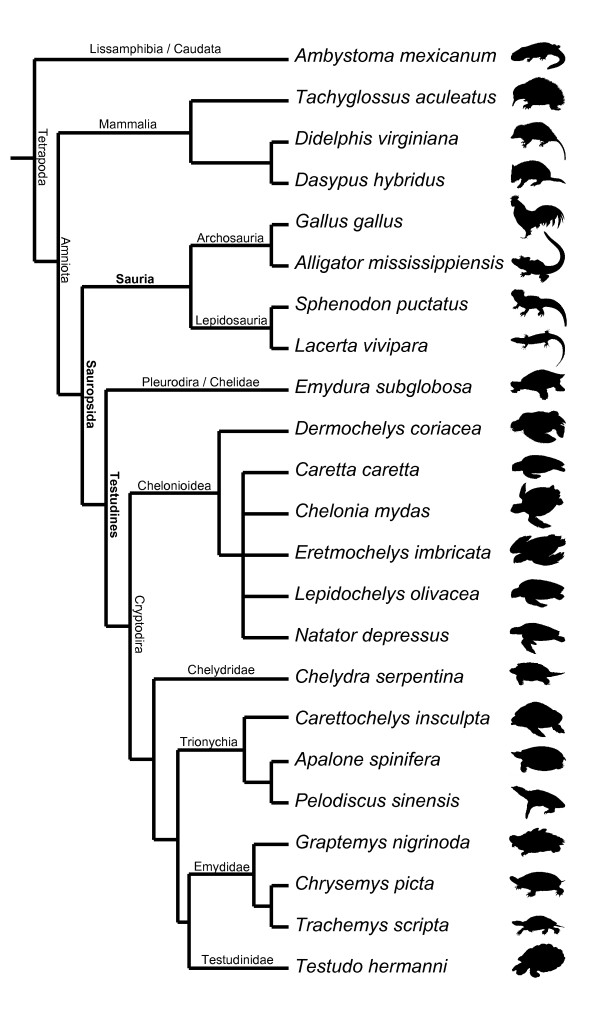
**Best supported topology of turtle relationships**. Best supported topology: turtles are the sister-group of all remaining living reptiles and sea turtles are basal within cryptodire turtles.

The turtle-saurian relationship is clearly the best supported hypothesis using Parsimov [19], independent of which hypothesis of inner turtle phylogeny is taken as reference. Among the latter, the cryptodire hypothesis with sea turtles in a basal position [6] was the best supported topology of all (56 consensus synapomorphies for unordered character states and 53 when using ordered character states, Figures 3 and 4). The Archosauria hypothesis has very little support in comparison to all main hypotheses considered (Figure 1, Table S6 and S7). The differences among the alternative phylogenies are distinctly significant and suggest that embryological data of the kind treated here are clearly most congruent with a basal position of turtles within Sauropsida.

As additional exploration of the developmental data on the alternative hypotheses, we mapped the event pairs on the 24 topologies and counted tree length for each of them. When mapping unordered character states, the tree lengths range between 6383 and 6429 (Table S6). The alternative tetrapod trees combined to one of the hypotheses of inner turtle relationship were the shortest ones (Figure 3). The best-supported topologies with 6383 steps are (Testudines + Sauria) and (Testudines + Lepidosauria). In contrast, when using the ordered character state approach, tree lengths range between 8886 and 8988 (Table S7). We found the shortest tree for a sister group position of turtles to the Sauria clade (Table S7) with 8886 steps. It is notable that both for the ordered and unordered character state approach the Testudines-Sauria tree is among the shortest and that the archosaurian hypothesis is among the most weakly supported ones (Table S6 and S7).

The analysis presented here clearly shows that heterochronic shifts during tetrapod development do occur and can be recognised even with the conservative Parsimov method [19,22]. The number of autapomorphies at a node on alternative phylogenies as test criterion is not a standard cladistic analysis, and the relative timings of events are not conventional characters either. But the differences among the alternative phylogenies are distinctly significant and suggest that embryological data of the kind treated here are clearly most congruent with a basal position of turtles within Sauropsida.

Characters that are phylogenetically informative are mostly those of the organogenic phase (from neurulation until more or less all organs are present). Character complexes such as eye, ear, eyelid, mandibular arch, pharyngeal arches, pharyngeal slits and neck flexure are involved in major temporal shifts. In contrast, we found few temporal shifts during limb development, in accordance with the conserved pattern across tetrapods reported in recent studies [23,24]. There are also few temporal shifts in the early period of embryonic development, that is, from the occurrence of blastopore and neurulation up to early somite cluster formation.

Some changes at the different nodes of the phylogenies examined are potentially linked with functional or ecological aspects. One character that clearly distinguishes Mammalia from Sauropsida is the temporal occurrence of the lens/iris contour. In mammals it occurs late with respect to the occurrence of several other events, in sauropsids earlier, perhaps coupled with the differing life strategies of these two groups. Since mammals are generally protected by and associated to the mother in the uterus or during the lactation period, they may not "need" to develop their eye as early as reptiles do, because the latter generally have to find food and avoid predators shortly after hatching. Other senses-related changes may also be interpretable in this light. Temporal shifts that characterise Testudines + Sauria are mainly related to the early occurrence of events of the eye, ear and nasal complex in relation to limb-related characters. The primary cervical flexure of 90° and its final disappearance is delayed within Sauropsida. This is possibly related to an earlier hatching/birth in mammals and their need for a muscular, neck-related suction. A late occurrence of the fourth pharyngeal slit in Sauria in relation to the occurrence of the third slit may be – compared to turtles as a sister group – correlated to a shorter neck. When discussing neck related characters also the count of cervical vertebrae and the ossification mode should be kept in mind. The Sauria are distinguished from the Testudines by an accelerated occurrence of some somite clusters. This is potentially coupled with the fact that whereas Testudines are characterised by a constant and low count of vertebrae [25], higher numbers of vertebrae are exhibited by saurian clades.

There are few empirical studies of sequence heterochrony with similar methods to those used here, so comparisons with our study are only preliminary. Other studies of a similar phylogenetic breadth [26,27] have found less heterochronies per node than reported here; studies at lower taxonomic levels and on diverse character complexes have found several heterochronic changes at different phylogenetic levels [20,28,29]. As claimed in recent comprehensive studies of skeletal development in mammals [20-22], heterochrony can be expressed in different ways (e.g., growth heterochrony [30]) and empirical studies are needed to further study its phylogenetic signal and relevance in vertebrate evolution.

Several extinct groups, for which no molecular or embryological data are available, have been suggested as potential sister-groups of turtles [31,32]. We cannot test based on our exclusively neontological data whether Testudines evolved within one of the fossil "Anapsida"-clades or on the stemline of Sauria within Diapsida [5]. Histological studies of head development should help understanding skull fenestration evolution and turtle origins. In this regard, the mode of jaw development in the snapping turtle *Chelydra serpentina *was preliminary interpreted as consistent with a stem-position of turtles outside of Diapsida [33].

## Conclusion

The embryological data and their analysis presented here are in agreement with a basal position of turtles instead of one nested within a saurian clade. Whatever future comprehensive genomic studies reveal, it is clear that important homoplasies have characterised either the evolution of adult morphological structures or the evolution of developmental patterns of organogenesis, or both, in land vertebrate evolution.

The elucidation of the developmental and molecular mechanisms [34,35] and the discovery of potentially transitional forms in the fossil record [7,36] for the turtle shell, in concern with solving the phylogenetic position of turtles, promises to explain one of the major evolutionary transitions in land vertebrate evolution.

## Methods

### Analyses of sequence heterochrony

The key methodology for a comparative analysis of developmental sequence data is 'event-pairing' [37]. It consists of the pair-wise comparison of events in a sequence, with each pair being assigned a score. The method permits to produce characters that can be analysed in the context of phylogeny. A matrix is created in which the occurrence of each element is related to every other one. Hence, each event-pair can be treated as a character with three possible character states reflecting the relative timing of one element relative to another: a. Event A occurs before event B (event-pair coded as 0). b. Event A occurs at the same time as event B (coded as 1). c. Event A occurs after event B (coded as 2). Published phylogenies can be used to plot the resulting characters. After this step the state at each node of the phylogeny can be inferred and the ancestral sequence reconstructed. *Parsimov *[19] is a method that is based on the basic event-pairing. It determines the minimal solution that accounts for every event-pair change and yields a consensus that contains all hypotheses of movement that must necessarily form part of any equally most parsimonious solution to the observed event-pair changes [[19]: page 239].

### Procedures implemented in the analysis

The procedures of the heterochrony analysis follow in general Jeffery et al. [19] with extensions by Olaf Bininda-Emonds (see below). Here the principal steps are summarised (find a detailed step-by-step-protocol in Table S5). First, a table listing species (columns) against characters (lines) was prepared. The stage-associated events were recorded for each species (Table S3). Afterwards the stages were temporally ranked for each species separately by the following principle: characters "A", "E", "F" occur first (rank 1), characters "B", "D", "G" occur second (rank 2), character "C", H" occur third (rank 3), etc. (Table S4). An event pair sequence for each species was generated (see Additional file 2 and 3) using the *perl*-based *EventpairSim.pl *and afterwards one topology was drawn with Mesquite 2.01 [38]. In PAUP* 4.0b10 [39] the event pairs were plotted over the unrooted topology by the principle of parsimony, the tree length was documented and ACCTRAN and DELTRAN character optimisations were calculated (Table S6). The heterochrony analyses were performed with the *perl*-based *parimv7g.pl *using Windows XP Pro SP2 on a 2.4 GHz computer; each run took approximately 20 hours when all tree analyses were running in parallel. The names of the events (Table S2) that were initially encoded as numbers for the analysis were retransformed to names at the end using *ReplacerParsimv.pl *[40]. The procedure was repeated for each of the tested proposed topologies separately (Table S6). For the preferred topology (Figures 4 and S3) autapomorphic heterochronic shifts are listed using unordered character states (Tables S8 and S9).

In addition to the Parsimov analyses and character mappings (Table S6 and S7) we did a phylogenetic reconstruction with PAUP* 4.0b10 (heuristic search) using event pairs as character states. Both for ordered and unordered characters no sensible cladogram results and turtles form a paraphyletic group (Figure S4 and S5). These results underline the worthlessness of the non independent event pairs for phylogenetic reconstructions [22].

### On the use of ordered vs. unordered characters

Whether to order characters or not in phylogenetic analyses is a controversial matter. In our analyses developmental ranks were encoded to produce event pairs comprising only three characters states [[19], see Methods: Analyses of sequence heterochrony]. We tested both the unordered and the ordered approaches. Ordering characters introduces additional assumptions, such as that a change or relative timing from 'late' to 'early' requires an intermediate step of simultaneity. But since developmental characters are of a continuous kind, ordering is a reasonable assumption [29,41,42]. Hence, we also ran the analyses with ordered characters to test the robusticity of our analyses (Table S7). The evidence for all supported topologies is the same. The topology Testudines + Sauria, with marine turtles basal within cryptodires, is the best supported. The second best supported hypothesis for the position of turtles within Tetrapoda is that of Testudines + Lepidosauria, followed by Testudines + Archosauria. In sum, both analyses together strongly support the same topology for the position of turtles and inner turtle phylogeny (Figures 3, 4 and S3).

### Treatment of event simultaneity

The issue of how to treat events scored as simultaneous has been discussed before [43-47]. Event simultaneity is generally ascribed to a lack of resolution because it is considered unlikely that two events occur at exactly the same time. However, perceptions of the impact of event simultaneity vary. Velhagen [43] cautioned against including simultaneous events in event pair analysis, arguing that these are nearly always artefactual. However, most event-pair analyses have included simultaneous event scores, often with a word of caution [19,39,44,48,49]. We follow Weisbecker et al. [22] in refraining from excluding characters that where coded as simultaneous, as we think that in our data set changes from simultaneity suggests in most cases real changes in timing and not lack of resolution. The alternative of treating simultaneous data as missing involves loss of information and introduces error and potentially spurious reconstructions of developmental change in phylogeny. For the sake of completeness we ran Parsimov for all topologies excluding the tie shifts (from 0 to 1, 1 to 2, 2 to 1, 1 to 0) and replaced all "1s" for "?" in the event pair matrix. We counted the consensus shifts for the branch leading to Testudines + sistergroup in each topology (Table S6 and S7) and listed the shifts overlapping in the ties-included and ties-excluded analyses (Tables S6 and S8).

### Taxonomic sampling

As an outgroup to all observed amniotes we have chosen the axolotl, *Ambystoma mexicanum*, based on detailed and comparative description of its developmental stages [50,51]. We refrained from taking a more basal lissamphibian species out of Gymnophiona [11] because members of this clade are morphologically highly derived [52], as shown by in the reduction of limbs. Comparative staging tables of Anura are published only for species characterised by several highly derived characters [53,54] and the development of their legs is switched around in comparison to the plesiomorphic vertebrate pattern [23]. Caudata show the plesiomorphic pattern of an early forelimb contra a late hind limb development. But compared to other caudates like *Necturus *[55] or *Andrias *[56] the axolotl obviously exhibits a delayed development of limbs during maturation [51]. When discussing the development of the limbs within Amniota this should be kept in mind.

Phylogenetic breadth in the sampling of published information was chosen based on the extent of existing descriptions and the quality of illustrations (Table S1). For the echidna, *Tachyglossus aculeatus*, the staging system of Semon [57] was used. To expand this information, we examined 21 embryo photographs and drawings of this species (Hubrecht collection, Berlin [58]) and ordered them chronologically by the number of somites (Table S3).

All taxa analysed are listed in Table S1. Each major tetrapod clade is represented by one species, except for Cryptodira, represented by 14 species (Figures 4 and S3). With this in mind we should be careful not to overinterpret character changes [29]. For example, our preliminary and cautious interpretation on the evolution of the neck length in Testudines vs. Sauria could be refuted by including pleurodire turtles with shorter necks (Pelomedusidae) or birds with elongated necks (Struthioniformes). Our examination of the embryology of sensory characters could be extended by including mammal and bird species of nidicolous as well as nidifugous behaviour. Also the ground pattern of neck length and flight behaviour in these groups should be addressed. When including taxa of different body shape the results could still remain similar to ours and the functional explanations should be rethought.

### Remarks on characters excluded from the heterochrony analysis

Examples of excluded characters are the shape and development of pigmentation, detailed carapace or scale differentiations – all characters important for staging prehatching embryos of a particular species. Although pigmentation and colouration are mentioned in most staging tables, some authors refer to first occurrence of pigment cells that are hard to see in photographs and in embryos that have been fixed for a long time. Other authors call an organ "pigmented" when the organ is completely "dark". In most cases we were not able to interpret the drawings and photographs as mentioned in the descriptions based on their different quality and the subjective view and approach of each author. We also refrained from listing every single somite number because there is high variability in the formation of the mesodermal segments. The presented definition of clusters, five somites each, serves to reflect the variance of somite development but is also an example of subjectivity in character definition – an unavoidable limitation of any morphological study of this kind.

In literature the forelimb development is usually better documented, because in general a more or less simultaneous development of fore- and hind limb is observable as we experienced when observing both limbs. When defining events for the limb development we either discuss both limbs separately or only the forelimb was discussed. If there had been an unrecorded "abnormal" temporal shift between fore- and hind limb in one particular species, it would have only reflected a derived feature of this species and would be useless for the approach of this study.

### Alternative hypotheses of cryptodire relationships

Whereas Gaffney and Meylan [59] and Shaffer et al. [60] hypothesised a basal position of the snapping turtles (Chelydridae) within Cryptodira (Figure 1a and S2a), Joyce's [6] analysis resulted in a basal position of the sea turtles (Chelonioidea) within living taxa (Figures 1b and S2b). Other authors [61,62] hypothesised the Trionychoidea (Kinosternidae + Trionychia) to be paraphyletic and placed Trionychia (soft-shelled turtles) basal to all remaining cryptodires. Trionychia are represented in our study (Figures 1c and S2c). The interrelationships of the Testudinoidea (swamp turtles and tortoises) and Trionychia species that we included in our analysis are widely accepted and follow Gaffney and Meylan [59]. Within Chelonioidea, *Dermochelys *is generally considered to be the sister group of all remaining marine turtle taxa [63]; given conflicts of morphological and molecular data [64], we set Chelonioidea excl. *Dermochelys *as unresolved for all analyses (Figures 4 and S3). Taxonomic names of turtles follow Fritz & Havaš [65].

## Authors' contributions

IW and MRS-V designed the research and wrote the paper, IW performed data collection and analyses.

## Supplementary Material

Additional file 1 **Supplementary Information**. Containing nine Tables (Tables S1–S9), five Figures (Figures S1–S5) and references to supplementary material. Click here for file

Additional file 2 **turtles.ep.nex**. Nexus data file containing the event paired matrix for all observed species (open i.e. with PAUP* or Mesquite software). Based on the rank table (Table S4), the event pairs were generated with eventpairsim.pl (see Table S5): The timing of two events (ranks) is compared among each other, generating an event pair: When event A occurs before B the event pair is coded as state "0". When event A and event B occur at the same time the event pair is coded as state "1" (simultaneity). When event A occurs after event B the event pair is coded as state "2". Click here for file

Additional file 3 **turtles.names.ep.nex**. Same matrix content as turtles.ep.nex. Instead of event pair numbers event names are listed (for event abbreviations compare Table S2). Click here for file
