# Supplementary material for: Timing of organogenesis support basal position of turtles in the amniote tree of life
Source: BMC Evol Biol. 2009 Apr 23;9:82. doi: 10.1186/1471-2148-9-82 (PMC2679012; doi:10.1186/1471-2148-9-82)
Supplement: Additional file 1 — Supplementary Information. Containing nine Tables (Tables S1–S9), five Figures (Figures S1–S5) and references to supplementary material. [file 1471-2148-9-82-S1.pdf]

Supplementary Information

As supplementary information this pdf file (nine associated Tables, five Figures and reference list) and two data files (event pair matrices) are available.

Content pdf file:

- **Table S1** Species used in this study.
- **Table S2** List of events observed.
- **Table S3** Relative timing of events and stages for species studied.
- **Table S4** Matrix listing the temporal ranks of the events for each species.
- **Table S5** Step-by-step protocol for a Parsimov analysis.
- **Table S6** Comparison of Parsimov results and tree lengths (PAUP mapping) for each tested topology (unordered characters).
- **Table S7** Comparison of Parsimov results and tree lengths (PAUP mapping) for each tested topology (ordered characters).
- **Table S8** List of temporal shifts that are autapomorphic for the nodes in the best-supported hypothesis (unordered characters).
- **Table S9** List of temporal shifts that are autapomorphic for the species in the best-supported hypothesis (unordered characters).
- **Figure S1** All hypotheses for the position of turtles within Tetrapoda summarised (extension of Figure 1).
- **Figure S2** Alternative hypotheses for the relationships of turtle subgroups (modified from Figure 1).
- **Figure S3** Best-supported topology (modified from Figure 4) including the number of nodes that are referred in Table S8.
- **Figure S4** Phylogentic reconstruction resulting from an event pair based PAUP* analysis using unordered characters.
- **Figure S5** Phylogentic reconstruction resulting from an event pair based PAUP* analysis using ordered characters.
- **References** References to supplementary information.

Online data files:

- **turtles.ep.nex** Nexus data file containing the event paired matrix for all observed species (open i.e. with PAUP* or Mesquite software). Based on the rank table (Table S4), the event pairs were generated with eventpair-sim.pl (see Table S5): The timing of two events (ranks) is compared among each other, generating an event pair: When event A occurs before B the event pair is coded as state “0”. When event A and event B occur at the same time the event pair is coded as state “1” (simultaneity). When event A occurs after event B the event pair is coded as state “2”.
- **turtles.names.ep.nex** Same matrix content as turtles.ep.nex. Instead of event pair numbers event names are listed (for event abbreviations compare Table S2).

Table S1 – Species used in this study

Main references, number of observed specimens and stages are listed.

No	Classification	species	references (<u>main references underlined</u>)	No. of observed specimens	described stages (followed staging table: stages)	blasto–porus+ [pictures] *
1	Caudata	***Ambystoma mexicanum***	1. <u>Bordzilovskaya et al. (1989)</u> [1] (stages 1–44),<br>2. <u>Nye et al. (2003)</u> [2] (stages 45–57)	n.d.	51 stages including "half stages" (Bordzilovskaya et al. 1989 [1]); 13 (Nye et al. 2003 [2])	42 [42] +13 [13]
2	Mammalia, Monotremata	***Tachyglossus aculeatus***	**<u>1. this study</u>** (stages IW1–13, Semon–54, –55)<br>2. <u>Semon (1894</u>a, b, <u>c</u>) [3–5](stages 40–53)	1. 22<br>2. n. d.	14 (this study: IW1–13, Semon–54), 14 (Semon 1894c [5]: 40–53)	13 [13] + 14 [14]
3	Mammalia, Theria, Marsupialia	***Didelphis virginiana***	<u>McCrandy (1938)</u> [6]	n.d.	35 (McCrandy 1938 [6]: 1–35)	20 [20]
4	Mammalia, Theria, Placentalia	***Dasypus hybridus (=Tatusia hybrida)***	<u>Fernandez (1915)</u> [7]	206+	35 (Fernandez 1915 [7]: 1–35)	24 [21]
5	Archosauria, Aves	***Gallus gallus***	<u>Hamburger & Hamilton (1951)</u> [8]	996+	45 (Hamburger & Hamilton 1951 [8]: 1–45)	44 [44]
6	Archosauria, Crocodylia	***Alligator mississippiensis***	<u>1. Ferguson (1985)</u> [9]<br>2. <u>Voeltzkow (1899)</u> [10]	1. 1500<br>2. n.d.	4 early stages (after Voeltzkow 1899 [10]), 28 (Ferguson 1985 [9]: 1–28)	32 [32]
7	Lepidosauria, Sphenodontida	***Sphenodon punctatus***	<u>1. Dendy (1899)</u> [11]<br>2. Moffat (1985) [12]	1. 90<br>2. n.d.	16 (Dendy 1899 [11]: C–S)	16 [15]
8	Lepidosauria, Squamata	***Lacerta vivipara***	<u>1. Dufaure & Hubert (1961)</u> [13]<br>2. Hubert (1985) (*42*)<br>3. Moffat (1985) (*40*)	1. 350<br>2. n.d.<br>3. n.d.	40 (Dufaure & Hubert 1961 [13]: 1–40)	36 [36]
9	Testudines, Cryptodira, Chelonioidea	***Caretta caretta***	1. Billett et al. (1992) [15]<br>2. <u>Miller (1985)</u> [16]<br>3. **this study**	1. n.d.<br>2. 1303<br>3. 5	31 (Miller 1985 [16]: 1–31)	22 [20]
10	Testudines, Cryptodira, Chelonioidea	***Chelonia mydas***	<u>1. Miller (1985)</u> [16]<br>2. Parker (1880) [17]<br>3. **this study**	1. 723<br>2. 26+<br>3. 18	31 (Miller 1985 [16]:1–31)	22 [19]
11	Testudines, Cryptodira, Chelonioidea	***Dermochelys coriacea***	<u>Renous et al. (1989)</u> [18]	97+	31 (Miller 1985 [16]: 1–31)	22 [22]
12	Testudines, Cryptodira, Chelonioidea	***Eretmochelys imbricata***	<u>Miller (1985)</u> [16]	567	31 (Miller 1985 [16]: 1–31)	22 [12]
13	Testudines, Cryptodira, Chelonioidea	***Lepidochelys olivacea***	<u>1. Crastz (1982)</u> [19]<br>2. **this study**	1. 210<br>2. 28	31 (Crastz 1982 [19]: 1–31)	31 [28]
14	Testudines, Cryptodira, Chelonioidea	***Natator depressus***	<u>Miller (1985)</u> [16]	375	31 (Miller 1985 [16]:1–31)	22 [10]
15	Testudines, Cryptodira, Chelydridae	***Chelydra serpentina***	**<u>Yntema (1968)</u>** [20]	Not noted	27 (Yntema 1968 [20]: 0–26)	27 [27]
16	Testudines, Cryptodira, Emydidae	***Chrysemys picta***	<u>Mahmoud et al. (1973)</u> [21]	446	23 (Mahmoud et al. 1973 [21]: 1–23)	23 [23]
17	Testudines, Cryptodira, Emydidae	***Graptemys nigrinoda***	**<u>this study</u>**	35	14 (Yntema 1968 [20]: 12–15, 17–26)	14 [14]
18	Testudines, Cryptodira, Emydidae	***Trachemys scripta***	<u>Greenbaum (2002)</u> [22]	104	15 (Yntema 1968 [20]: 12–26)	15 [15]
19	Testudines, Cryptodira, Testudinidae	***Testudo hermanni***	<u>Guyot et al. (1994)</u> [23]	161	27 (Yntema 1968 [20]: 0/1–26)	27 [27]
20	Testudines, Cryptodira, Trionychia	***Apalone spinifera***	<u>Greenbaum & Carr (2002)</u> [24]	112	15 (Yntema 1968 [20]: 12–26)	15 [14]
21	Testudines, Cryptodira, Trionychia	***Carettochelys insculpta***	<u>Beggs et al. (2000)</u> (*53*)	54	15 (Yntema 1968 [20]: 12–26)	15 [15]
22	Testudines, Cryptodira, Trionychia	***Pelodiscus sinensis***	<u>1. Tokita & Kuratani (2001)</u> [26]<br>2. **this study**	1. 67<br>2. 6	23 (Tokita & Kuratani 2001 [26]: 5–27)	23 [23]
23	Testudines, Pleurodira	***Emydura subglubosa***	**this study**	18	14 (Yntema 1968 [20]: 12–15, 17–26)	14 [14]

*** documented stages since blastopore"stage" and number of related pictures (photographs or drawings)**

Table S2 – List of events observed in this study

Nomenclature follows Werneburg [27]. Abbreviations as used in event paired matrix (online data suppl.) and in the following tables are marked in bold letters. A standard event code is presented in a progressional format summarising all used character complexes and characters evolved in Vertebrata (V), Gnathostomata (G), Tetrapoda (T), Amniota (A) and Sauropsida (S).

Clade that shows character complex	standard code for character complex	character complex	standard event code for character	Number of used character	Character (event)
Vertebrata (V)	V01	egg	V01a	1	**egg lay**
	V02	blastula	V02a	2	**blastopore** visible
	V03	neural tube	V03a	3	**primitive streak** visible
			V03b	4	**neural folds** begin **closure**
			V03c	5	**anterior neuropore** is **closed**
			V03d	6	**posterior neuropore** is **closed**
	V04	somites	V04a	7	**somites** are **hard** to **count** from now on
			V04b	8	**1–5 somite pairs** visible
			V04c	9	**6–10 somite pairs** visible
			V04d	10	**11–15 somite pairs** visible
			V04e	11	**16–20 somite pairs** visible
			V04f	12	**21–25 somite pairs** visible
			V04g	13	**26–30 somite pairs** visible
			V04h	14	**31–35 somite pairs** visible
			V04i	15	**36–40 somite pairs** visible
			V04j	16	**41–45 somite pairs** visible
			V04k	17	**46–50 somite pairs** visible
	V05	head	V05a	18	**head** distinct as a **bulbus**
			V05b	19	**anterior cephalic projection** visible
			V05c	20	**head projection disappeared**
	V06	nose	V06a	21	**olfactory pit** visible
			V06b	22	**external nares** visible
	V07	ear	V07a	23	**otic pit** visible
			V07b	24	**otic vesicle** visible
			V07c	25	**otic capsule** became **inconspicuous**
	V08	eye	V08a	26	**optic vesicle** visible
			V08b	27	**lens vesicle** visible
			V08c	28	**optic fissure** visible
			V08d	29	**contour lens/iris** visible
			V08e	30	**pupil forms**
			V08f	31	**scleral papillae** visible
			V08g	32	**scleral papillae** became **inconspicuous**
	V09	ribs	V09a	33	**rib primordia** visible
	V10	heart	V10a	34	**ventricle bulbus** visible
			V10b	35	**thoracal bulbus disappeared**
			V10c	36	**ventricle S-shaped**
	V11	tail	V11a	37	**tail bud** visible
	V12	limbs	V12a	38	**forelimb ridge** visible
			V12b	39	**forelimb bud** visible
			V12c	40	**forelimb elongated**
			V12d	41	**forelimb AER** visible
			V12e	42	**hindlimb AER** visible
			V12f	43	**forelimb elbow** visible
			V12g	44	**forelimb paddle** visible
			V12h	45	**hindlimb paddle** visible
			V12i	46	**forelimb digital plate** visible
			V12j	47	**hindlimb digital plate** visible
			V12k	48	**digital grooves** visible
			V12l	49	**digital serration** visible

			V12m	50	first **finger** visible
			V12n	51	**first claw** visible
	V13	scales/etc.	V13a	52	**head scales** visible
			V13b	53	**throat scales** visible
			V13c	54	**eyelid scales** visible
			V13d	55	**neck scales** visible
			V13e	56	**back scales** visible
			V13f	57	**limb scales** visible
			V13g	58	**whole forelimb** covered by **scales**
			V13h	59	**tail scales** visible
			V13i	60	**carapace** scutes (**scales**) visible
	V14	hatch	V14a	61	**hatch**ing
Gnathostomata (G)	G01	maxillary process	G01a	62	**max**illary process (**bud**) visible
			G01b	63	**max**illary process at **posterior** border of the **eye**
			G01c	64	**max**illary process at **midline** of the **eye**
			G01d	65	**max**illary process at **anterior** border of the **lens**
			G01e	66	**max**illary process at **anterior** border of the **eye**
			G01f	67	**max**illary process and the **frontonasal** process **fuse**
	G02	mandibular process	G02a	68	**mand**ibular **arch** visible as a **bud**
			G02b	69	**mand**ibular process at **posterior** border of the **eye**
			G02c	70	**mand**ibular process at **posterior** border of the **lens**
			G02d	71	**mand**ibular process at **midline** of the **eye**
			G02e	72	**mand**ibular process at **anterior** border of the **lens**
			G02f	73	**mand**ibular process at **anterior** border of the **eye**
			G02g	74	**mand**ibular process at the **level** of the **frontonasal** process
			G02g	75	**mand**ibular process at the level of the **occlusion point**
	G03	pharyngeal arches	G03a	76	**2nd** pharyngeal **arch** visible
			G03b	77	**3rd** pharyngeal **arch** visible
			G03c	78	**4th** pharyngeal **arch** visible
			G03d	79	**5th** pharyngeal **arch** visible
			G03e	80	**hyoid flap** formed on 2nd pharyngeal arch
	G04	pharyngeal slits	G04a	81	**1st** pharyngeal **slit** visible
			G04b	82	**2nd** pharyngeal **slit** visible
			G04c	83	**3rd** pharyngeal **slit** visible
			G04d	84	**4th** pharyngeal **slit** visible
			G04e	85	pharyngeal **slits closed**
	G05	urogenital papillae	G05a	86	**urogenital papilla bud** occurs
			G05b	87	**urogenital papilla** became **inconspicuous**
Tetrapoda (T)	T01	neck	T01a	88	**cervical flexure** of **90°** occurs
			T01b	89	**cervical flexure disappeared**
			T01c	90	**wrinkles** visible **on neck**
Amniota (A)	A01	eye lids	A01a	91	**lower lid** visible
			A01b	92	**eyelid** has **begun overgrow**ing the eye
			A01c	93	**eyelid at** the lower level of the **scleral papillae**
			A01d	94	**eyelid** at the **ventral** border of the **lens**
			A01e	95	**eyelid** covers at least **half** of the **eye**
			A01f	96	**membrana nictitans** visible
	A02	caruncle	A02a	97	**caruncle** visible
Sauropsida (S)	S01	rhamphothecae	S01a	98	**rhamphothecae** visible
	S02	carapace	S02a	99	**carapacial ridge** visible
			S02b	100	**longitudinal carapacial ridge** visible
			S02c	101	**carapace not** clearly limited **anterior**ly
			S02d	102	**carapace clearly limited**
			S02e	103	**carapace** projects **beyond tail** root
			S02f	104	**carapace** became **irregular**

Table S3 – Relative timing of events and stages for species studied

First number: stage as described in original reference (see Table S1); underlined numbers: named after drawings/photographs; *italicised blue numbers*: filled in using information from references (Yntema 1968 [20] and Miller 1985 [16]) as proposed by the cited authors.

Name of event	standard event code for character	Number of used character	*Ambystoma mexicanum*	*Tachyglossus aculeatus*	*Didelphis virginiana*	*Dasypus hybridus*	*Gallus gallus*	*Alligator mississippiensis*	*Sphenodon punctatus*	*Lacerta vivipara*	*Caretta caretta*	*Chelonia mydas*	*Dermochelys coriacea*	*Eretmochelys imbricata*	*Lepidochelys olivacea*	*Natator depressus*	*Chelydra serpentina*	*Chrysemys picta*	*Graptemys nigrinoda*	*Trachemys scripta*	*Testudo hermanni*	*Apalone spinifera*	*Carettochelys insculpta*	*Pelodiscus sinensis*	*Emydura subglubosa*
egg lay	V01a	1	1	12	?	?	1	1	"B"	1	*6*	*6*	*6*	*6*	1	*6*	0	*0*	*0*	*0*	0–1	*0*	*0*	*1*	*0*
blastopore	V02a	2	10	5	16	?	2	?	C	5	*6*	6	*6*	*6*	1	*6*	0	1	?	?	0–1	?	?	?	?
primitive streak	V03a	3	13	6	17	12	4	*-4*	E	*12*	*10*	10	10	*10*	*3*	*10*	*4*	3	?	?	3	?	?	*4*	?
neural folds closure	V03b	4	18	?	25	16	8	-3	G	18	*13*	*13*	11	*13*	3–4	*13*	4	5	?	?	5	?	?	6	?
anterior neuropore closed	V03c	5	19	18	24	20	12	-2	K	20	*14*	*14*	12	*14*	4	*14*	7	8	?	?	7	?	?	8	?
posterior neuropore closed	V03d	6	18	12	25	20	17	-1	J	24	?	15	14	15	7?	?	5	7	?	?	9	?	?	7	?
somites hard count	V04a	7	35	19	31	29	22	6	?	31	*21*	*21*	20	*21*	*14*	*21*	12	15	14	14	13	13	16	13	15
1–5 somite pairs	V04b	8	22	8	23	18	8	?	?	17	*11*	*11*	10	*11*	*3–4*	*11*	5	5	?	?	5	?	?	6	?
6–10 somite pairs	V04c	9	26	9	?	19	10	1	?	21	*12*	*12*	13	*12*	*4*	*12*	7	7	?	?	7	?	?	12	?
11–15 somite pairs	V04d	10	28	11	25	20	11	1	J	24	*14*	*14*	14	*14*	*5*	*14*	8	8	?	?	?	?	12	9	?
16–20 somite pairs	V04e	11	32	12	26	?	13	1	?	25	*15*	*15*	15	*15*	*7–8*	*15*	9	9	?	?	8	?	15	?	?
21–25 somite pairs	V04f	12	34	14	?	24	15	2	K	26	*16*	*16*	16	*16*	*8–9*	*16*	10	13	?	?	9	?	?	10	12
26–30 somite pairs	V04g	13	?	16	?	?	17–18	3	L	?	*17*	*17*	17	*17*	*9–10*	*17*	?	14	?	?	10	?	?	12	?
31–35 somite pairs	V04h	14	?	?	?	26	18	4	?	28	*18*	*18*	18	*18*	*10*	*18*	11	?	?	?	11	?	?	?	13
36–40 somite pairs	V04i	15	?	18	?	?	19–20	5	?	29	*18*	*18*	18	*18*	*10*	*18*	?	?	13	?	12	?	?	?	14
41–45 somite pairs	V04j	16	?	?	?	28	?	?	M	?	*19*	*19*	19	*19*	*11*	*19*	?	?	?	?	?	?	?	?	?
46–50 somite pairs	V04k	17	?	?	?	?	?	?	?	30	?	?	?	?	?	?	?	?	?	?	?	?	?	?	?
head bulbus	V05a	18	21	?	24	19	9	–2	H–J	21	12	11	11	12	4	11	6	6	?	?	5	?	?	6	?
anterior cephalic projection	V05b	19	27	7	26	20	13	1	K	25	13	13	12	13	6	13	7	7	?	?	6	?	?	7	?
head projection disappeared	V05c	20	?	23	34	35	36	24	R	42*	26	31	26	26	20	26	21	22	?	?	20	21	20–21	22–23	20
olfactory pit	V06a	21	26	15	?	25	?	6	L	?	16	?	17	*17*	?	*17*	10	9	?	13	12	13	19	10	?
external nares	V06b	22	29	17	28	28	?	7	M	26	18	16	18	*23*	18	*23*	13	11	12	*25*	13	*24*	20	16	12
otic pit	V07a	23	23	13	24	19	?	1	H	25	?	*12*	12	*12*	5	*12*	7	8	?	16	?	?	17	8	12
otic vesicle	V07b	24	26	?	28	20	12	2	K	26	14	*13*	13	*13*	8	*13*	9	9	12	?	8	?	18	10	12
otic capsule inconspicuous	V07c	25	35	24	?	?	?	?	O	?	*18*	20	17	18	14	18	14	11	17	?	11	?	23	12	15
optic vesicle	V08a	26	21	15	24	19	9	1	H	21	13	*11*	12	*11*	?	*11*	7	6	?	?	6	?	?	7	?
lens vesicle	V08b	27	29	18	?	25	14	9	K	26	16	*14*	13	*14*	?7	*14*	9	9	12	?	9	?	?	9	?
optic fissure	V08c	28	?	15	?	30	25	12	L	27	*16*	17	16	*16*	?	*16*	?	9	13	?	11	?	14?	10	12
contour lens/iris	V08d	29	37	22	33	31	26	10	L	27	*16*	*16*	15	*16*	11	*16*	12	15	14	14	13	15	14	10	12
pupil forms	V08e	30	37	?	?	30	31	13	N	32	?	21	17	?	13	?	13	?	14	14	14	17	14	13	14
scleral papillae	V08f	31	?	?	?	?	30	?	Q	34	*24*	19	24	*24*	?	*24*	16	19	18	18	16	16	16	17	19
scleral papillae inconspicuous	V08g	32	?	?	?	?	37	?	?	36	?	23	27	?	?	?	20	20	21	19	20	21	21	20	22
rib primordia	V09a	33	?	?	?	?	?	17	?	30	*23*	*23*	?	*23*	16	*23*	?	?	17	16	?	?	14	16	?
Ventricle bulbus	V10a	34	31	15	26	20	9	1	J	22	13	*9*	13	*9*	4	*9*	8	7	?	?	7	?	?	7	?
thoracal bulbus disappeared	V10b	35	38	22	33	35	34	22	P	36	25	24	24	24	17	24	17	19	17	19	18	19	18	18	17
ventricle S–shaped	V10c	36	?	?	?	?	12	1	J	?	*13*	*13*	*13*	*13*	5–6	*13*	?	8	?	?	?	?	?	8	?
tail bud	V11a	37	22	14	27	23	17	3	L	22	14	*16*	15	*16*	7	*16*	10	9	?	?	8	?	?	8	?
forelimb ridge	V12a	38	38	14	26	25	17	6	?	27	16	17	16	16	9	16	9	10	12	12	10	12	?	10	12
forelimb bud	V12b	39	43	15	?	27	23	9	M	29	?	18	18	?	10	?	11–12	11	?	?	11	?	?	12	13
forelimb elongated	V12c	40	46	17	28	28	24	10	?	30	19	19	19	?	12	?	12	12	13	13	12	13	12	13	14
forelimb AER	V12d	41	?	?	28	?	23	10	?	30	20	18	20	*20*	?	*20*	12	14	13	?	12	12	?	12	14

hindlimb AER	V12e	42	?	?	28	?	22	9	?	30	20	20	20	20	?	20	12	14	14	?	12	12	?	13	14
forelimb elbow	V12f	43	51	?	32	?	25	13	N	32	20	20	21	20	12	20	14	15	14	18	14	16	15	13	14
forelimb paddle	V12g	44	?	17	29	28	25	11	N	31	19	19	?	19	12	19	14	12	13	14	13	?	12	12	13
hindlimb paddle	V12h	45	?	17	32	28	24	10	O	31	19	?	21	19	13	19	14	12	13	?	?	?	14	?	14
forelimb digital plate	V12i	46	48	19	30	29	24	14	O	32	20	21	21	18	?	18	14	13	14	15	14	14	14	13	15
hindlimb digital plate	V12j	47	54	20	32	29	25	14	P	32	20	21	21–22	18	?	18	14	15	14	15	14	14	14	14	15
digital grooves	V12k	48	48	20	30–31	29	25	16	?	33	23	23	23	23	13	23	16	16	?	16	16	16	16	16	?
digital serrati-on	V12l	49	48	20	31	30	27	18	Q	33	23	24	24	25	?	24	17	18	?	17	17	17	13	17	?
finger	V12m	50	51	22	33	33	32	20	R	36	25	?	?	?	?	?	19	19	20	19	18	19	21	20	18
first claw	V12n	51	?	28	34	35	36	23	?	36	25	25	?	25	21	25	21	21	20	23	21	20	20	21	19
head scales	V13a	52	?	x	x	x	31	20	R	37	26	25	26	26	23	27	22	?	24	22	20	21	23	22	21
throat scales	V13b	53	?	x	x	x	30	23	R	37	26	26	26	?	24	27	21	?	24	22	22	21	22	24–25	21
eyelid scales	V13c	54	?	x	x	x	?	23	R	40	26	25	26	25	23	26	22	?	24	22	?	21	24	22	?
neck scales	V13d	55	?	x	x	x	30	20	R	36	26	26	26	26	20	27	18	20	24	22	18	21	24	22	18
back scales	V13e	56	?	x	x	x	31	18	R	35	?	?	?	?	?	?	?	?	?	?	?	?	?	?	?
limb scales	V13f	57	?	x	x	x	30	22	R	37	25	25	26	25	23	26	21	20	22	20	20	21	22	21	21
whole fore-limb scales	V13g	58	?	x	x	x	?	23	R	39	26	26–27	26	26–27	26	27	22	22	24	22	21	21	23	23	21
tail scales	V13h	59	?	x	x	x	31	21	Q	35	26	26	26	?	25	27	19	23	24	21	22	21	?	?	21
carapace scutes	V13i	60	?	x	x	x	?	?	?	?	25	24	26	25	?	24	?	18	20	15	18	18	19	19	18
hatch	V14a	61	41	24	x	x	46	28	T	40	31	31	31	31	31	31	26	26	26	26	26	26	26	27	26
max bud	G01a	62	40	15	27	24	18–19	9	M	29	18	16	17	18	9	18	12	9	?	?	6	?	?	?	?
max posterior eye	G01b	63	?	15	27–28	24	22	?	M	29	?	?	17	?	10	?	11	?	?	?	8	11	?	11	?
max midline eye	G01c	64	40	15–16	28	25	23	10	O	30	18	?	?	?	11	?	12	10	12	?	12	?	?	12	12
max anterior lens	G01d	65	41	16	29	26	24	12	?	31	19	17	19	18	12	18	?	?	?	12	?	?	?	13	13
max anterior eye	G01e	66	?	17	30	27	?	10	P	?	20	19	20	24	13	24	13	?	?	13	13	13	13	13	14
max frontona-sal fuse	G01f	67	42	22	31	33	28	14	Q	33	21	23	23	?	17	?	14	?	14	14	16	14	14	14	15
mand arch bud	G02a	68	20	9	27	23	14	1	M	24	16	13	13	13	11	13	5	7	?	?	6	?	?	8	?
mand posterior eye	G02b	69	21	18	28	?	?	?	M	28	18	18	16	18	13	18	15	10	12	?	?	14	15	?	12
mand posterior lens	G02c	70	35	?	?	?	27	13	?	30	22	23	?	?	16	?	16	?	14	12	15	15	16	14	13
mand midline eye	G02d	71	41	19	29	24	29	16	N	31	24	24	19	24	?	?	?	?	?	14	?	?	?	?	14
mand anterior lens	G02e	72	?	20	?	25	30	?	?	33	25	?	20	25	16	19	17	17	17	?	?	16	17	16	15
mand anterior eye	G02f	73	?	21	29–30	26	31	17	?	34	25	25	?	?	17	?	?	18	20	15	17	?	17–18	17	?
mand level frontonasal	G02g	74	42	22	32	27	32	18	?	35	25	25	24	26	20	?	18	19	21	16	18	17	18	20	17
mand occlusi-on point	G02g	75	42	25	34	34–35	33	20	R	38	25–26	26	24	26	22	25–26	19	19–20	23	18	18–19	18	21	22	17
2nd arch	G03a	76	24	12	27	24	14	2	M	25	16	13	15	13	11	13	7	9	13	12	6	12	12	10	12
3rd arch	G03b	77	25	15	27	24	15	4	M	27	16	17	15	?	?	?	8	9	?	12	8	11	13	11	12
4th arch	G03c	78	25	15	27	25	?	8	N	30	18	17	16	?	?	?	10	11	13	12	9	11	?	11	12
5th arch	G03d	79	28	?	?	?	?	9	N	?	?	17	17	?	?	?	11	12	?	12	9	11	?	11	12
hyoid flap	G03e	80	41	?	?	?	24	10	?	?	20	19	?	18	?	18	11	12?	?	14	?	?	14	14	15
1st slit	G04a	81	23	12	27	?	14	2	K	25	16	15	16	15	8	15	10	9	13	12	9	11	?	9	12
2nd slit	G04b	82	27	12	27	?	14	?	L	26	19	16	16	16	8	16	10	10	?	12	10	11	?	10	12
3rd slit	G04c	83	31	15	28	?	15	9	L	28	19	17	16	17	?	17	?	10	13	12	10	?	?	11	12
4th slit	G04d	84	?	15	?	?	18–19	?	?	30	19	17	17	17	?	17	?	11	13	12	?	?	?	13	12
slits closed	G04e	85	55*	20	30	?	26	14	Q	34	22	23	24	19	9	19	12	12	14	15	14	?	?	15	17
urogenital papilla bud	G05a	86	31	22	29	?	?	8	?	31	19	18	21	18	12	18	?	17	13	12	14	13	16	13	13
urogenital papilla in-conspicuous	G05b	87	?	28	35	?	?	?	?	?	28	28	25–26	28	?	28	?	20	?	21	?	23	?	?	21
cervical flexure 90°	T01a	88	?	x	28	25	18	5	M	23	21	18	18	21	12	17	13	15	12	13	12	13	?	8	17
cervical flexure disap-peared	T01b	89	29	x	34	30	33	23	?	36	31	31	25	31	15	31	25	21	21	21	21	26	26	22	26
wrinkles on neck	T01c	90	?	x	35	?	?	21	R	35	24	25	25	25	22	24	20	20	21	21	20	19	18	22	?
lower lid	A01a	91	x	21	31	31	28	19	Q	33	24	23	24	22	18	22	18	20	17	18	19	18	18	15	17
eyelid begun overgrow	A01b	92	x	22	?	33	36	21	R	37	25	24	27	25	?	?	19	21	18	19	20	18	20	16	17
eyelid at scleral papillae	A01c	93	x	?	?	33	36	?	?	38	26	25	27	26	?	26	?	?	18	19	20	19	20	19	?
eyelid ventral lens	A01d	94	x	23	33	34	37	21	R	39	28	28	29	27	?	28	20	22–23	21	20	23	20	21	23	17
eyelid half eye	A01e	95	x	24	34	35	38	25	?	40	29	30	29	28	?	?	23	?	23	23	24	22	25	24	24

membrana nictitans	A01f	96	x	?	?	?	34	19	?	?	?	28	28?	27	?	27	?	?	25	20	22	20	?	23	?
caruncle	A02a	97	x	22	x	x	30	16	R	34	*24*	23	24	*24*	18	*24*	17	18	17	17	18	18	?	17	17
rhamphothecae	S01a	98	x	x	x	x	x	x	x	x	24	26	24	*23*	?	*23*	19	19	20	16	19	16	?	19	20
carapacial ridge	S02a	99	x	x	x	x	x	x	x	x	21	21	21	*21*	14	*21*	14	14	15	15	14	14	14	14	15
longitudinal carapacial ridge	S02b	100	x	x	x	x	x	x	x	x	22	22	23	?	15	?	15	15	?	?	15	15	15	15	?
carapace not anterior	S02c	101	x	x	x	x	x	x	x	x	24	22	23	24	16	23	?	15	?	?	?	?	15	15	?
carapace clearly limited	S02d	102	x	x	x	x	x	x	x	x	25	23	24	25	?22	24	16	16	17	16	16	16	16	16	17
carapace beyond tail	S02e	103	x	x	x	x	x	x	x	x	25	23	24	24	18	24	20	18	17	19	18	19	17	17	17
carapace irregular	S02f	104	x	x	x	x	x	x	x	x	25	24	?	25	21	26	17	15	18	17	?	19	19	22	21

Table S4 – Matrix listing the temporal ranks of the events for each species

Note that stages of the kind "21–22" (see Table S4) are encoded as separate ranks.

name of event	standard event code	number of event	*Ambystoma_mexicanum*	*Tachyglossus_aculeatus*	*Didelphis_virginiana*	*Dasypus_hybridus*	*Gallus_gallus*	*Alligator_mississippiensis*	*Sphenodon_punctatus*	*Lacerta_vivipara*	*Caretta_caretta*	*Chelonia_mydas*	*Dermochelys_coriacea*	*Eretmochelys_imbricata*	*Lepidochelys_olivacea*	*Natator_depressus*	*Chelydra_serpentina*	*Chrysemys_picta*	*Graptemys_nigrinoda*	*Trachemys_scripta*	*Testudo_hermanni*	*Apalone_spinifera*	*Carettochelys_insculpta*	*Pelodiscus_sinensis*	*Emydura_subglobosa*
**egg lay**	**V01a**	**1**	1	7	?	?	1	5	1	1	1	1	1	1	1	1	1	1	1	1	1	1	1	1	1
**blastopore**	**V02a**	**2**	2	1	1	?	2	?	2	2	1	1	1	1	1	1	1	2	?	?	1	?	?	?	?
**primitive streak**	**V03a**	**3**	3	2	2	1	3	1	3	3	2	3	2	3	2	3	2	3	?	?	2	?	?	2	?
**neural folds closure**	**V03b**	**4**	4	?	5	2	4	2	4	5	5	6	3	6	3	6	2	4	?	?	3	?	?	3	?
**anterior neuropore closed**	**V03c**	**5**	5	14	4	5	8	3	8	6	6	7	4	7	4	7	5	7	?	?	5	?	?	5	?
**posterior neuropore closed**	**V03d**	**6**	4	7	5	5	12	4	7	10	?	8	6	8	8	?	3	6	?	?	7	?	?	4	?
**somites hard count**	**V04a**	**7**	19	15	15	12	17	10	?	17	13	14	12	14	18	14	11	14	4	4	11	4	6	10	5
**1–5 somite pairs**	**V04b**	**8**	8	4	3	3	4	?	?	4	3	4	2	4	3	4	3	4	?	?	3	?	?	3	?
**6–10 somite pairs**	**V04c**	**9**	12	5	?	4	6	5	?	7	4	5	5	5	4	5	5	6	?	?	5	?	?	9	?
**11–15 somite pairs**	**V04d**	**10**	14	6	5	5	7	5	7	10	6	7	6	7	5	7	6	7	?	?	?	?	2	6	?
**16–20 somite pairs**	**V04e**	**11**	17	7	6	?	9	5	?	11	7	8	7	8	9	8	7	8	?	?	6	?	5	?	?
**21–25 somite pairs**	**V04f**	**12**	18	9	?	7	11	6	8	12	8	9	8	9	11	9	8	12	?	?	7	?	?	7	2
**26–30 somite pairs**	**V04g**	**13**	?	12	?	?	13	7	9	?	9	10	9	10	13	10	?	13	?	?	8	?	?	9	?
**31–35 somite pairs**	**V04h**	**14**	?	?	?	9	14	8	?	14	10	11	10	11	14	11	9	?	?	?	9	?	?	?	3
**36–40 somite pairs**	**V04i**	**15**	?	14	?	?	16	9	?	15	10	11	10	11	14	11	?	?	3	?	10	?	?	?	4
**41–45 somite pairs**	**V04j**	**16**	?	?	?	11	?	?	10	?	11	12	11	12	15	12	?	?	?	?	?	?	?	?	?
**46–50 somite pairs**	**V04k**	**17**	?	?	?	?	?	?	?	16	?	?	?	?	?	?	?	?	?	?	?	?	?	?	?
**head bulbus**	**V05a**	**18**	7	?	4	4	5	3	6	7	4	4	3	5	4	4	4	5	?	?	3	?	?	3	?
**anterior cephalic projection**	**V05b**	**19**	13	3	6	5	9	5	8	11	5	6	4	6	7	6	5	6	?	?	4	?	?	4	?
**head projection disappeared**	**V05c**	**20**	?	19	18	17	30	27	15	27	19	23	19	19	23	20	20	22	?	?	19	12	12	20	9
**olfactory pit**	**V06a**	**21**	12	10	?	8	?	10	9	?	8	?	9	10	?	10	8	8	?	3	10	4	10	7	?
**external nares**	**V06b**	**22**	15	13	9	11	?	11	10	12	10	9	10	16	22	16	12	10	2	14	11	15	11	13	2
**otic pit**	**V07a**	**23**	9	8	4	4	?	5	5	11	?	5	4	5	5	5	5	7	?	6	?	?	7	5	2
**otic vesicle**	**V07b**	**24**	12	?	10	5	8	6	8	12	6	6	5	6	10	6	7	8	2	?	6	?	9	7	2
**otic capsule inconspicuous**	**V07c**	**25**	19	20	?	?	?	?	12	?	10	13	9	11	18	11	13	10	6	?	9	?	15	9	5
**optic vesicle**	**V08a**	**26**	7	10	4	4	5	5	5	7	5	4	4	4	?	4	5	5	?	?	4	?	?	4	?
**lens vesicle**	**V08b**	**27**	15	14	?	8	10	13	8	12	8	7	5	7	8	7	7	8	2	?	7	?	?	6	?
**optic fissure**	**V08c**	**28**	?	10	?	13	20	16	9	13	8	10	8	9	?	9	?	8	3	?	9	?	4	7	2
**contour lens/iris**	**V08d**	**29**	20	18	17	14	21	14	9	13	8	9	7	9	15	9	11	14	4	4	11	6	4	7	2
**pupil forms**	**V08e**	**30**	20	?	?	13	26	17	11	18	?	14	9	?	17	?	12	?	4	4	12	8	4	10	4
**scleral papillae**	**V08f**	**31**	?	?	?	?	25	?	14	20	16	12	16	17	?	17	15	18	7	8	14	7	6	14	8
**scleral papillae inconspicuous**	**V08g**	**32**	?	?	?	?	31	?	?	22	?	16	20	?	?	?	19	20	9	9	19	12	13	17	11
**rib primordia**	**V09a**	**33**	?	?	?	?	?	20	?	16	15	16	?	16	20	16	?	?	6	6	?	?	4	13	?
**Ventricle bulbus**	**V10a**	**34**	31	10	6	5	5	5	7	8	5	2	5	2	4	2	6	6	?	?	5	?	?	4	?
**thoracal bulbus disappeared**	**V10b**	**35**	21	18	17	17	29	25	13	22	17	17	16	17	21	17	16	18	6	9	16	10	9	15	6
**ventricle S–shaped**	**V10c**	**36**	?	?	?	?	8	5	7	?	5	6	5	6	6	6	?	7	?	?	?	?	?	5	?
**tail bud**	**V11a**	**37**	8	9	7	6	12	7	9	8	6	9	7	9	8	9	8	8	?	?	6	?	?	5	?
**forelimb ridge**	**V12a**	**38**	21	9	6	8	12	10	?	13	8	10	8	9	12	9	7	9	2	2	8	3	?	7	2
**forelimb bud**	**V12b**	**39**	26	10	?	10	18	13	10	15	?	11	10	?	14	?	10	10	?	?	9	?	?	9	3
**forelimb elongated**	**V12c**	**40**	27	13	9	11	19	14	?	16	11	12	11	?	16	?	11	11	3	3	10	4	2	10	4
**forelimb AER**	**V12d**	**41**	?	?	9	?	18	14	?	16	12	11	12	13	?	13	11	13	3	?	10	3	?	9	4
**hindlimb AER**	**V12e**	**42**	?	?	9	?	17	13	?	16	12	13	12	13	?	13	11	13	4	?	10	3	?	10	4
**forelimb elbow**	**V12f**	**43**	29	?	16	?	20	17	11	18	12	13	13	13	16	13	13	14	4	8	12	7	5	10	4
**forelimb paddle**	**V12g**	**44**	?	13	11	11	20	15	11	17	11	12	?	12	16	12	13	11	3	4	11	?	2	9	3
**hindlimb paddle**	**V12h**	**45**	?	13	16	11	19	14	12	17	11	?	13	12	17	12	13	11	3	?	?	?	4	?	4
**forelimb digital plate**	**V12i**	**46**	28	15	13	12	19	18	12	18	12	14	13	11	?	11	13	12	4	5	12	5	4	10	5
**hindlimb digital plate**	**V12j**	**47**	30	16	16	12	20	18	13	18	12	14	14	11	?	11	13	14	4	5	12	5	4	11	5
**digital grooves**	**V12k**	**48**	28	16	14	12	20	19	?	19	15	16	15	16	17	16	15	15	?	6	14	7	6	13	?
**digital serration**	**V12l**	**49**	28	16	15	13	22	21	14	19	15	17	16	18	?	17	16	17	?	7	15	8	3	14	?
**finger/toe**	**V12m**	**50**	29	18	17	15	27	23	15	22	17	?	?	?	?	?	18	18	8	9	16	10	13	17	7
**first claw**	**V12n**	**51**	?	22	18	17	30	26	?	22	17	18	?	18	24	18	20	21	8	13	20	11	11	18	8
**head scales**	**V13a**	**52**	?	?	?	?	26	23	15	23	19	18	19	19	26	21	21	?	12	12	19	12	15	19	10
**throat scales**	**V13b**	**53**	?	?	?	?	25	26	15	23	19	19	19	?	27	21	20	?	12	12	21	12	14	23	10
**eyelid scales**	**V13c**	**54**	?	?	?	?	?	26	15	26	19	18	19	18	26	20	21	?	12	12	?	12	16	19	?
**neck scales**	**V13d**	**55**	?	?	?	?	25	23	15	22	19	19	19	19	23	21	17	20	12	12	16	12	16	19	7
**back scales**	**V13e**	**56**	?	?	?	?	26	21	15	21	?	?	?	?	?	?	?	?	?	?	?	?	?	?	?
**limb scales**	**V13f**	**57**	?	?	?	?	25	25	15	23	17	18	19	18	26	20	20	20	10	10	19	12	14	18	10
**whole forelimb scales**	**V13g**	**58**	?	?	?	?	?	26	15	25	19	20	19	20	29	21	21	22	12	12	20	12	15	21	10
**tail scales**	**V13h**	**59**	?	?	?	?	26	24	14	21	19	19	19	?	28	21	18	24	12	11	21	12	?	?	10
**carapace scales**	**V13i**	**60**	?	?	?	?	?	?	?	?	17	17	19	18	?	17	?	17	8	5	16	9	10	16	7
**hatch**	**V14a**	**61**	24	20	?	?	33	29	16	26	22	23	23	23	30	23	24	25	14	15	24	16	18	24	13
**max bud**	**G01a**	**62**	22	10	7	7	15	13	10	15	10	9	9	11	12	11	11	8	?	?	4	?	?	?	?
**max posterior eye**	**G01b**	**63**	?	10	8	7	17	?	10	15	?	?	9	?	14	?	9	?	?	?	6	2	?	8	?
**max midline eye**	**G01c**	**64**	23	11	9	8	18	14	12	16	10	?	?	?	15	?	11	9	2	?	10	?	?	9	2
**max anterior lens**	**G01d**	**65**	24	12	11	9	19	16	?	17	11	10	11	11	16	11	?	?	?	2	?	?	?	10	3
**max anterior eye**	**G01e**	**66**	?	13	13	10	?	14	13	?	12	12	12	17	17	17	12	?	?	3	11	4	3	10	4
**max frontonasal fuse**	**G01f**	**67**	25	18	15	15	23	18	14	19	13	16	15	?	21	?	13	?	4	4	14	5	4	11	5
**mand arch bud**	**G02a**	**68**	6	5	7	6	10	5	10	10	8	6	5	6	15	6	3	6	?	?	4	?	?	5	?
**mand posterior eye**	**G02b**	**69**	7	14	9	?	?	?	10	14	10	11	8	11	17	11	14	9	2	?	?	5	5	?	2
**mand posterior lens**	**G02c**	**70**	19	?	?	?	22	17	?	16	14	16	?	?	20	?	15	?	4	2	13	6	6	11	3
**mand midline eye**	**G02d**	**71**	24	15	11	7	24	19	11	17	16	17	11	17	?	?	?	?	?	4	?	?	?	?	4
**mand anterior lens**	**G02e**	**72**	?	16	?	8	25	?	?	19	17	?	12	18	20	12	16	16	6	?	?	7	7	13	5
**mand anterior eye**	**G02f**	**73**	?	17	12	9	26	20	?	20	17	18	?	?	21	?	?	17	8	5	15	?	8	14	?
**mand level frontonasal**	**G02g**	**74**	25	18	16	10	27	21	?	21	17	18	16	19	23	?	17	18	9	6	16	8	9	17	6
**mand occlusion point**	**G02g**	**75**	25	21	18	16	28	23	15	24	18	19	16	19	25	19	18	19	11	8	17	9	13	19	6
**2nd arch**	**G03a**	**76**	10	7	7	7	10	6	10	11	8	6	7	6	15	6	5	8	3	2	4	3	2	7	2
**3th arch**	**G03b**	**77**	11	10	7	7	11	8	10	13	8	10	7	?	?	?	6	8	?	2	6	2	3	8	2
**4th arch**	**G03c**	**78**	11	10	7	8	?	12	11	16	10	10	8	?	?	?	8	10	3	2	7	2	?	8	2
**5th arch**	**G03d**	**79**	14	?	?	?	?	13	11	?	?	10	9	?	?	?	9	11	?	2	7	2	?	8	2
**hyoid flap**	**G03e**	**80**	24	?	?	?	19	14	?	?	12	12	?	11	?	11	9	11	?	4	?	?	4	11	5
**1st slit**	**G04a**	**81**	9	7	7	?	10	6	8	11	8	8	8	8	10	8	8	8	3	2	7	2	?	6	2
**2nd slit**	**G04b**	**82**	13	7	7	?	10	?	9	12	11	9	8	9	10	9	8	9	?	2	8	2	?	7	2
**3th slit**	**G04c**	**83**	16	10	9	?	12	13	9	14	11	10	8	10	?	10	?	9	3	2	8	?	?	8	2
**4th slit**	**G04d**	**84**	?	10	?	?	15	?	?	16	11	10	9	10	?	10	?	10	3	2	?	?	?	10	2
**slits closed**	**G04e**	**85**	31	16	13	?	22	18	14	20	14	16	16	12	12	12	11	11	4	5	12	?	?	12	6
**urogenital papilla bud**	**G05a**	**86**	16	18	11	?	?	12	?	17	11	11	13	11	16	11	?	16	3	2	12	4	6	10	3
**urogenital papilla inconspicuous**	**G05b**	**87**	?	22	19	?	?	?	?	?	20	21	18	22	?	22	?	20	?	11	?	14	?	?	10
**cervical flexure 90°**	**T01a**	**88**	?	?	9	8	14	9	10	9	13	11	10	14	16	10	12	14	2	3	10	4	?	5	6
**cervical flexure disappeared**	**T01b**	**89**	15	?	18	13	28	26	?	22	22	23	17	23	19	23	23	21	9	11	20	16	18	19	13
**wrinkles on neck**	**T01c**	**90**	?	?	19	?	?	24	15	21	16	18	17	18	25	17	19	20	9	11	19	10	9	19	?
**lower lid**	**A01a**	**91**	?	17	15	14	23	22	14	19	16	16	16	15	22	15	17	20	6	8	18	9	9	12	6
**eyelid begun overgrow**	**A01b**	**92**	?	18	?	15	30	24	15	23	17	17	20	18	?	?	18	21	7	9	19	9	11	13	6
**eyelid at scleral papillae**	**A01c**	**93**	?	?	?	16	30	?	?	24	19	18	20	19	?	20	?	?	7	9	19	10	11	16	?
**eyelid ventral lens**	**A01d**	**94**	?	19	17	16	31	24	15	25	20	21	22	21	?	22	19	23	9	10	22	11	13	21	6
**lower half eye**	**A01e**	**95**	?	20	18	17	32	28	?	26	21	22	22	22	?	?	22	?	11	13	23	13	17	22	12
**membrana nictitans**	**A01f**	**96**	?	?	?	?	29	22	?	?	?	21	21	21	?	21	?	?	13	10	21	11	?	21	?
**caruncle**	**A02a**	**97**	?	18	?	?	25	19	15	20	16	16	16	17	22	17	16	17	6	7	16	9	?	14	6
**rhamphothecae**	**S01a**	**98**	?	?	?	?	?	?	?	?	16	19	16	16	?	16	18	18	8	6	18	7	?	16	9
**carapacial ridge**	**S02a**	**99**	?	?	?	?	?	?	?	?	13	14	13	14	18	14	13	13	5	5	12	5	4	11	5
**longitudinal carapacial ridge**	**S02b**	**100**	?	?	?	?	?	?	?	?	14	15	15	?	19	?	14	14	?	?	13	6	5	12	?
**carapace not anterior**	**S02c**	**101**	?	?	?	?	?	?	?	?	16	15	15	17	20	16	?	14	?	?	?	?	5	12	?
**carapace clearly limited**	**S02d**	**102**	?	?	?	?	?	?	?	?	17	16	16	18	25	17	15	15	6	6	14	7	6	13	6
**carapace beyond tail**	**S02e**	**103**	?	?	?	?	?	?	?	?	17	16	16	17	22	17	19	17	6	9	16	10	7	14	6
**carapace irregular**	**S02f**	**104**	?	?	?	?	?	?	?	?	17	17	?	18	24	20	16	14	7	7	?	10	10	19	10

Table S5 – Step-by-step protocol for a Parsimov analysis

Protocol after Jeffery et al. [28] with extensions by Olaf R. P. Bininda-Emonds. Special thanks to Janine M. Ziermann (University of Leiden) who introduced us to details of Parsimov – her PhD-thesis [29] represents a fundament for cognition and an extended case study using Parsimov method. **Bold**: **letters to type in** / CAPITAL LETTERS: BUTTON IN PROGRAM / ***Bold italic: data and software files*** / *Italic letters: additional information* / _: space tab on keyboard.

No.	Instructions
1	Prepare an Excel-table **(*stages.xls*)** listing species (first column) against characters (first line). List for each species, which event occurs at which stage (see our Table S3). For species names there must not be a free space between genera and species names.
2	Generate an Excel file listing numbers of characters (events) in the first column and the name of these characters in the second column. Save as txt file **(*names.txt*)**.
3	Based on ***stages.xls***, rank the stages temporally – each species separately – by the following principle: In the first species: characters “A”, “B”, “C” occur first (rank 1), characters “D”, “E”, “F” occur second (rank 2), character “G”, “H” occur third (rank 3). In a second species the character sequence is different: characters “A”, “E”, “F” occur first (rank 1), characters “B”, “D”, “G” occur second (rank 2), character “C”, H” occur third (rank 3), etc. (see our Table S4). Save as xls file (***ranks.xls***).
4	Eliminate the first line (the event/character names) of ***ranks.xls*** because in the following steps only species/numbers lines are used by the programs. Information on event/character names does not get lost and in step 14 they will be replaced. Save this as a new file, ***turtles.txt*** (*tabstop txt file*), in a folder C:\analysis.
5	Installation of the software perl (www.perl.org), Mesquite 2.01. [30], PAUP* 4.0b10 (PC or Mac) [31].
6	Put the following applications into the C:\analysis folder: ***EventPairSim.pl***, ***Parsimv7g.pl*** [28] and ***ReplacerParsimv.pl*** (all available on [32]). Put also ***ranks.xls*** and ***names.txt*** into this folder.
7	Type into the command prompt of the PC the following command: **cd_c:\analysis** (*Enter, note that “_” means space tab on keyboard*) – **perl_EventPairSim.pl_turtles.txt_23_104** *(Enter)*. The first number, 23, is the number of taxa, the second the number, 104, of events used in this study. This procedure uses software ***perl*** and ***EventPairSim.pl*** and the file ***turtles.txt***.
8	Following Smith [33] a perl based event pairing is done by the program now, where the temporal occurrence of each event is compared to each other and coded as follows: “0” – event X occurs earlier than event Y in development; “1” – events X and Y occur at the same time; “2” – event X occurs later than Y. A new file, ***turtles.ep.nex***, where an event pair sequence (for details see Jeffery et al. [28]) is computed for each single species, is saved by ***EventPairSim.pl*** in C:\analysis (find our file ***turtles.ep.nex*** in online supplement). Check: Open the nex file with Mesquite or PAUP* software. You will see an event-paired sequence each species. E.g. find in our file: Taxon15Chelydraserpentina1222212222222202… etc.
9	In the following step you can arrange your proposed topology of taxa in the ***turtle.ep.nex*** file. Using Mesquite and the following clicks: TAXA&TREES – NEW TREE WINDOW – STORED TREE – *(now arrange branches with the “move branch” function*) –TREE – STORE TREE – FILE – SAVE FILE.
10	Character optimisations (Acctran, Deltran). You have two alternatives, MAC or Windows. We used PAUP (Windows) for our analyses:<br>a. When using PAUP* (Mac): Now open the branch arranged ***turtle.ep.nex*** file in PAUP (MAC) and do as follows: FILE – OPEN (***turtle.ep.nex***) – EDIT – YES: NEW FILE *(not: deroot)* – FILE – EXECUTE – first unroot the tree: therefore scroll down in the PAUP*-text and you find: tree NAME = [&R] („,); end**;** here you have to displace R *(rooted tree)* by U *(unrooted tree)*. Now you have: tree NAME = [&U] („,); end; – FILE – EXECUTE

	– ANALYSIS – PARSIMONY SETTINGS – CHARACTER OPTIMISATIONS – ACCTRAN – FILE –LOG OUTPUT TO DISK *(now record starts)* – YES: NEW FILE – TREE – DESCRIBE TREE – LIST OF APOMORPHIES – DESCRIBE – ***Acctran.log*** (*file name*) – FILE –LOG OUTPUT TO DISK *(record is stopped)* – repeat the procedure for the deltran optimisation: FILE – OPEN (***turtle.ep.nex***) – EDIT – YES: NEW FILE *(not: deroot)* – FILE – EXECUTE – first unroot the tree: therefore scroll down in the PAUP*-text and you find: tree NAME = [&**R**] (,,,); end**;** here you have to displace **R** *(rooted tree)* by **U** *(unrooted tree)*. Now you have: tree NAME = [&**U**] (,,,); end; – FILE – EXECUTE – ANALYSIS – PARSIMONY SETTINGS – CHARACTER OPTIMISATIONS – DELTRAN – FILE –LOG OUTPUT TO DISK *(now record starts)* – YES: NEW FILE – TREE – DESCRIBE TREE – LIST OF APOMORPHIES – DESCRIBE – ***Deltran.log*** (*file name*) – FILE –LOG OUTPUT TO DISK *(record is stopped)*<br>b. When using PAUP* (Windows): Start PAUP software and do the following: open ***turtle.ep.nex*** – *asking you, unroot?* type: **y** *(unroots the tree)* – **set_opt_=_acctran** – **showtree** – **showmatrix** – FILE – LOG OUTPUT TO DISK – SAVE AS ***Acctran.log*** – **describetree/apolist=yes** – FILE – LOG OUTPUT TO DISK – repeat the whole procedure as follows: open ***turtle.ep.nex*** *(Enter) – asking you, unroot?* type: **y** *(yes, Enter)* – **set_opt_=_deltran** *(Enter)* – **showtree** *(Enter)* – **showmatrix** *(Enter)* – FILE – LOG OUTPUT TO DISK – SAVE AS ***Deltran.log*** – **describetree/apolist=yes** *(Enter)* – FILE – LOG OUTPUT TO DISK
11	Now two files are generated and saved in C:\analysis: ***Acctran.log*** and ***Deltran.log***. These files can be opened with Notepad or Word.
12	Parsimov analysis. Type into the command prompt of the PC the following command: **cd_c:\analysis** *(Enter)* – **perl_parsimv7g.pl_Acctran.log** *(Enter)* – **m** *(manual, Enter)* – **Acctran.log** *(Enter)* – **a** *(all, Enter)* – **y** *(yes, Enter)* – **n** *(no, Enter)* – **turtleParsimovAcctran** *(future filename, Enter) – repeat the whole procedure for the deltran.log file:* Type into the command prompt of the PC the following command: **cd_c:\analysis** *(Enter)* – **perl_parsimv7g.pl_Deltran.log** *(Enter)* – **m** *(manual, Enter)* – **Deltran.log** *(Enter)* – **a** *(all, Enter)* – **y** *(yes, Enter)* – **n** *(no, Enter)* – **turtleParsimovDeltran** *(future filename, Enter*)
13	Depending on extend of data and the speed of the computer the analyses may take hours. At the end you get two files: ***turtleParsimovAcctran.crk*** and ***turtleParsimovDeltran.crk***. These can be opened with Wordpad or Word. You can see numbers representing the temporal shifts: i.e. character 23 moved E *(Early)* in respect to characters 15, 54, 76, 101.
14	At the end the numbers can be replace by the event names that were previously defined. This step uses ***replacerparsimv.pl*** and ***names.txt*** (see step 2). Follow this procedure: Change the file name from ***turtleParsimovAcctran.crk*** to ***turtleParsimovAcctran.crk.txt***. Type into the command prompt of the PC the following command: **cd_c:\analysis** *(Enter)* – **perl_replacerparsimv.pl_turtleParsimovAcctran.crk.txt_names.txt** *(enter)* – **m** *(manual, Enter)* – **turtleParsimovAcctran.crk.txt** *(file to use, Enter)* **–** **names.txt** *(definition file, Enter)* – repeat the whole procedure for the ***turtleParsimovAcctran.crk*** file and follow this procedure: Change the file name from ***turtleParsimovDeltran.crk*** to ***turtleParsimovDeltran.crk.txt***. Type into the command prompt of the PC the following command: **cd_c:\analysis** *(Enter)* – **perl_replacerparsimv.pl_turtleParsimovDeltran.crk.txt_names.txt** *(enter)* – **m** *(manual, Enter)* – **turtleParsimovDeltran.crk.txt** *(file to use, Enter)* **–** **names.txt** *(definition file, Enter)*
15	Now the numbers are replaced by the event names in the two files.

Table S6 – Comparison of Parsimov results and tree length (unordered characters)

Parsimov results and tree lengths (PAUP* analyses) for the tested hypotheses of the turtle origin within Tetrapoda (A–H) and the relationships of turtle crown taxa (a–c, compare Figure 1, Figure S1 and S2). Best-supported trees in bold type. Parsimov: Consensus (CON), Acctran (ACC), Deltran (DEL) of the ties-including analyses (ties = "incomplete" shifts from "0" to "1" or "2" to "1"). The first number in brackets is the count of consensus shifts in a ties-excluded analysis. The second number in brackets is the count of consensus shifts in both the ties-including and ties-excluding analyses. PAUP*: Consistency index (CI), homoplasy index (HI), CI excluding uninformative characters (CIe), HI excluding uninformative characters (HIe), Retention index (RI), Rescaled consistency index (RC) are listed. The Sauria + Testudines hypothesis ("C") combined to the cryptodire "b" hypothesis is the one with the highest Parsimov support. Note that the position of turtles next to all remaining amniotes (tree 1–3) could not be tested with Parsimov due to the use of only one outgroup (*Ambystoma mexicanum*): the direction of heterochronic shifts leading either to the outgroup or to the ingroup is not detectable. References for each hypothesis listed below.

amniote phylogeny (compare Figure S1 and S2)		cryptodire phylogeny (basal taxa shown)		Tree	Parsimov results	PAUP* (mapping)	
						Tree length	Tree Indices
A)	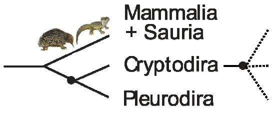	a	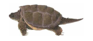	1	Not applicable	6388	CI = 0.5038, HI = 0.4962, CIe = 0.4070, Hie = 0.5930, RI = 0.2181, RC = 0.1098
		b	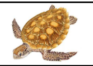	2	Not applicable	6390	CI = 0.5036, HI = 0.4964, CIe = 0.4069, HIe = 0.5931, RI = 0.2176, RC = 0.1096
		c	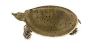	3	Not applicable	6394	CI = 0.5033, HI = 0.4967, CIe = 0.4066, HIe = 0.5934, RI = 0.2166, RC = 0.1090
B)	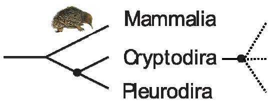	a	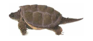	4	8 (7/7) / 68 / 18	6385	CI = 0.5040, HI = 0.4960, CIe = 0.4073, HIe = 0.5927, RI = 0.2188, RC = 0.1103
		b	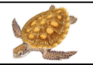	5	5 (8/4) / 64 / 16	6390	CI = 0.5036, HI = 0.4964, CIe = 0.4069, HIe = 0.5931, RI = 0.2176, RC = 0.1096
		c	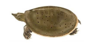	6	0 (9/0) / 51 / 16	6392	CI = 0.5034, HI = 0.4966, CIe = 0.4067, HIe = 0.5933, RI = 0.2171, RC = 0.1093
C)	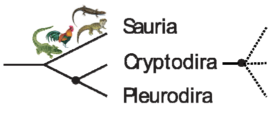	a	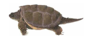	7	51 (38/28) / 118 / 80	**6383**	CI = 0.5042, HI = 0.4958, CIe = 0.4074, HIe = 0.5926, RI = 0.2193, RC = 0.1106
		b	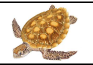	8	**56 (64/35) / 131 / 81**	6386	CI = 0.5039, HI = 0.4961, CIe = 0.4072, HIe = 0.5928, RI = 0.2185, RC = 0.1101
		c	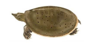	9	44 (37/27) / 107 / 72	6391	CI = 0.5035, HI = 0.4965, CIe = 0.4068, HIe = 0.5932, RI = 0.2173, RC = 0.1094
D)	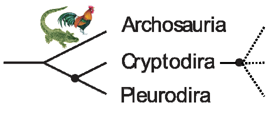	a	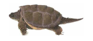	10	20 (9/7) / 55 / 27	6390	CI = 0.5036, HI = 0.4964, CIe = 0.4069, HIe = 0.5931, RI = 0.2176, RC = 0.1096
		b	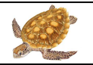	11	18 (9/7) / 48 / 23	6394	CI = 0.5033, HI = 0.4967, CIe = 0.4066, HIe = 0.5934, RI = 0.2166, RC = 0.1090
		c	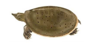	12	20 (7/7) / 45 / 27	6402	CI = 0.5027, HI = 0.4973, CIe = 0.4060, HIe = 0.5940, RI = 0.2146, RC = 0.1079
E)	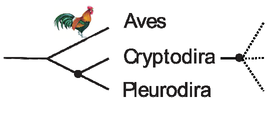	a	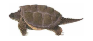	13	3 (7/0) / 38 / 16	6418	CI = 0.5014, HI = 0.4986, CIe = 0.4048, HIe = 0.5952, RI = 0.2107, RC = 0.1056
		b	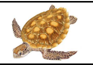	14	6 (9/1) / 62 / 18	6419	CI = 0.5013, HI = 0.4987, CIe = 0.4047, HIe = 0.5953, RI = 0.2104, RC = 0.1055
		c	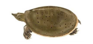	15	0 (7/0) / 0 / 13	6429	CI = 0.5005, HI = 0.4995, CIe = 0.4039, HIe = 0.5961, RI = 0.2079, RC = 0.1041
F)	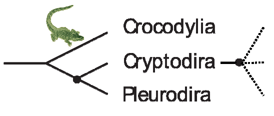	a	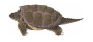	16	4 (2/2) / 58 / 26	6404	CI = 0.5025, HI = 0.4975, CIe = 0.4058, HIe = 0.5942, RI = 0.2141, RC = 0.1076
		b	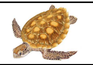	17	3 (4/1) / 27 / 20	6408	CI = 0.5022, HI = 0.4978, CIe = 0.4055, HIe = 0.5945, RI = 0.2131, RC = 0.1070
		c	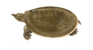	18	6 (3/1) / 52 / 29	6413	CI = 0.5018, HI = 0.4982, CIe = 0.4051, HIe = 0.5949, RI = 0.2119, RC = 0.1063
G)	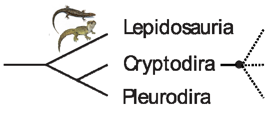	a	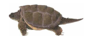	19	27 (23/13) / 70 / 50	**6383**	CI = 0.5042, HI = 0.4958, CIe = 0.4074, HIe = 0.5926, RI = 0.2193, RC = 0.1106
		b	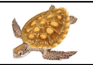	20	26 (24/13) / 62 / 48	6385	CI = 0.5040, HI = 0.4960, CIe = 0.4073, HIe = 0.5927, RI = 0.2188, RC = 0.1103
		c	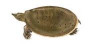	21	22 (20/14) / 53 / 51	6392	CI = 0.5034, HI = 0.4966, CIe = 0.4067, HIe = 0.5933, RI = 0.2171, RC = 0.1093
H)	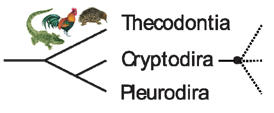	a	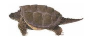	22	8 (0/0) / 45 / 39	6384	CI = 0.5041, HI = 0.4959, CIe = 0.4073, HIe = 0.5927, RI = 0.2190, RC = 0.1104
		b	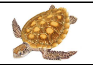	23	11 (0/0) / 38 / 39	6390	CI = 0.5036, HI = 0.4964, CIe = 0.4069, HIe = 0.5931, RI = 0.2176, RC = 0.1096
		c	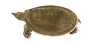	24	10 (0/0) / 41 / 42	6391	CI = 0.5035, HI = 0.4965, CIe = 0.4068, HIe = 0.5932, RI = 0.2173, RC = 0.1094

Reference numbers for Amniota hypothesis A) [34–36], for position of Diadectomorpha compare: [37], **hypothesis B)** [38], **hypothesis C)** [39–50], **hypothesis D)** [40: Tree–2, 51–62], **hypothesis E)** [63–65], **hypothesis F)** [66: Tree–3, 67–68], **hypothesis G)** [69–78], **hypothesis H)** [79–81]**,** Thecodontia = Crocodylia + (Aves + Mammalia), the term Thecodontia as only used by [79–81], **References for Cryptodira hypothesis a)** [39, 82], **hypothesis b)** [83–84], **hypothesis c)** [85] **– All hypotheses are summarised and discussed in detail by Rieppel [41, 86] and Scheyer [60].**

Table S7 – Comparison of Parsimov results and tree length (ordered characters)

Parsimov results and tree lengths (PAUP* analyses) for the tested hypotheses of the turtle origin within Tetrapoda (A–H) and the relationships of turtle crown taxa (a–c, see Figure 1, Figure S1 and S2). Best-supported tree in bold type. Parsimov: Consensus (CON), Acctran (ACC), Deltran (DEL) of the ties-including analyses (ties = "incomplete" shifts from "0" to "1" or "2" to "1"). PAUP*: Consistency index (CI), homoplasy index (HI), CI excluding uninformative characters (CIe), HI excluding uninformative characters (HIe), Retention index (RI), Rescaled consistency index (RC) are listed. The Sauria + Testudines hypothesis ("C") combined to the cryptodire "b" hypothesis is the one with the highest Parsimov support (tree No. 8). Note that the position of turtles next to all remaining amniotes (tree 1–3) could not be tested with Parsimov due to the use of only one outgroup (*Ambystoma mexicanum*): the direction of heterochronic shifts leading either to the outgroup or to the ingroup is not detectable. For references and illustrations of hypothesis tested, see Table S6.

Tree	Parsimov results	PAUP* (mapping)	
		Tree length	Tree Indices
1	Not applicable	8888	CI = 0.4245, HI = 0.5755, CIe= 0.3444, HIe = 0.6556, RI = 0.2418, RC = 0.1026
2	Not applicable	8902	CI = 0.4238, HI = 0.5762, CIe = 0.3438, HIe = 0.6562, RI = 0.2397, RC = 0.1016
3	Not applicable	8896	CI = 0.4241, HI = 0.5759, CIe = 0.3440, HIe = 0.6560, RI = 0.2406, RC = 0.1020
4	7 / 61 / 25	8891	CI = 0.4244, HI = 0.5756, CIe = 0.3443, HIe = 0.6557, RI = 0.2413, RC = 0.1024
5	21 / 102 / 26	8900	CI = 0.4239, HI = 0.5761, CIe = 0.3439, HIe = 0.6561, RI = 0.2400, RC = 0.1017
6	14 / 91 / 21	8903	CI = 0.4238, HI = 0.5762, CIe = 0.3437, HIe = 0.6563, RI = 0.2395, RC = 0.1015
7	49 / 122 / 64	**8886**	CI = 0.4246, HI = 0.5754, CIe = 0.3445, HIe = 0.6555, RI = 0.2421, RC = 0.1028
8	**53 / 130 / 68**	8892	CI = 0.4243, HI = 0.5757, CIe = 0.3442, HIe = 0.6558, RI = 0.2412, RC = 0.1023
9	41 / 120 / 46	8895	CI = 0.4242, HI = 0.5758, CIe = 0.3441, HIe = 0.6559, RI = 0.2407, RC = 0.1021
10	15 / 70 / 23	8912	CI = 0.4234, HI = 0.5766, CIe = 0.3433, HIe = 0.6567, RI = 0.2382, RC = 0.1009
11	16 / 62 / 32	8918	CI = 0.4231, HI = 0.5769, CIe = 0.3431, HIe = 0.6569, RI = 0.2373, RC = 0.1004
12	16 / 60 / 35	8925	CI = 0.4227, HI = 0.5773, CIe = 0.3428, HIe = 0.6572, RI = 0.2363, RC = 0.0999
13	4 / 63 / 36	8968	CI = 0.4207, HI = 0.5793, CIe = 0.3409, HIe= 0.6591, RI = 0.2299, RC = 0.0967
14	9 / 54 / 38	8972	CI = 0.4205, HI = 0.5795, CIe = 0.3407, HIe = 0.6593, RI = 0.2293, RC = 0.0964
15	8 / 58 / 36	8988	CI = 0.4198, HI = 0.5802, CIe = 0.3400, HIe = 0.6600, RI = 0.2269, RC = 0.0953
16	18 / 71 / 33	8946	CI = 0.4218, HI = 0.5782, CIe = 0.3419, HIe = 0.6581, RI = 0.2332, RC = 0.0983
17	18 / 62 / 33	8956	CI = 0.4213, HI = 0.5787, CIe = 0.3414, HIe = 0.6586, RI = 0.2317, RC = 0.0976
18	18 / 56 / 33	8958	CI = 0.4212, HI = 0.5788, CIe = 0.3413, HIe = 0.6587, RI = 0.2314, RC = 0.0975
19	38 / 88 / 55	8894	CI = 0.4242, HI = 0.5758, CIe = 0.3441, HIe = 0.6559, RI = 0.2409, RC = 0.1022
20	42 / 90 / 63	8894	CI = 0.4242, HI = 0.5758, CIe = 0.3441, HIe = 0.6559, RI = 0.2409, RC = 0.1022
21	31 / 85 / 54	8905	CI = 0.4237, HI = 0.5763, CIe = 0.3437, HIe = 0.6563, RI = 0.2393, RC = 0.1014
22	13 / 58 / 36	8891	CI = 0.4244, HI = 0.5756, CIe = 0.3443, HIe = 0.6557, RI = 0.2413, RC = 0.1024
23	14 / 53 / 41	8895	CI = 0.4242, HI = 0.5758, CIe = 0.3441, HIe = 0.6559, RI = 0.2407, RC = 0.1021
24	14 / 58 / 42	8903	CI = 0.4238, HI = 0.5762, CIe = 0.3437, HIe = 0.6563, RI = 0.2395, RC = 0.1015

Table S8 – Temporal shifts for nodes (unordered characters)

List of temporal shifts that are autapomorphic for the nodes in the best-supported hypothesis (Sauria+Testudines/Cryptodira“a”). First character occurs L=late or E=early in relation to the second character(s). For shifts charactersing Testudines and Sauropsida (Testudines + Sauria) asterisks mark ties (“incomplete” shifts from “0” to “1” or “2” to “1”). For Sauropsida consensus shifts are marked by asterisks that occur both in the tie including analysis (as presented for all remaining taxa) and in the analysis excluding tie shifts (so called “complete shifts”). For referred nodes compare with Figure S3. For abbreviations, see Table S2.

shift no.	branch	standard event code …	character no. …	character …	… moved LATE (L) / EARLY (E) in relation to …	… characters (CONSENSUS)
11	Amniota (Node 41) → Mammalia (Node 25)	V08d	29	contour lens/iris	**L**	optic fissure, digital grooves, max anterior lens, mand midline eye
58	Mammalia (Node 25) → Theria (Node 24)	G02a	68	mand arch bud	**L**	11–15 somite pairs, optic vesicle, tail bud
63		G03a	76	2nd arch	**L**	max bud, 3rd arch
120	Amniota (Node 41) → Sauropsida (Node 40)		0			Twins (anterior cephalic projection, otic pit)
121		A02a	97	anterior neuropore closed	**E**	26–30 somite pairs*, 36–40 somite pairs
123		V04g	13	26–30 somite pairs	**E**	forelimb bud*, max midline eye*, max anterior lens
127		V06b	22	external nares	**E**	forelimb AER, hindlimb AER, forelimb paddle, hindlimb paddle
128		V07b	24	otic vesicle	**E**	tail bud*, forelimb AER*, hindlimb AER*, mand posterior eye*, 1st slit
129		V07c	25	otic capsule inconspicu-ous	**E**	forelimb digital plate, lower lid
130		V08b	27	lens vesicle	**E**	mand posterior eye*
132		V08d	29	contour lens/iris	**E**	forelimb paddle*, mand posterior eye*, lower lid
134		V10a	34	ventricle bulbus	**E**	11–15 somite pairs*, 16–20 somite pairs*, tail bud*, forelimb ridge*, 2nd arch*, 1st slit*, 2nd slit*
136		V12b	39	forelimb bud	**E**	max anterior lens*
137		V12f	43	forelimb elbow	**E**	forelimb digital plate*, digital grooves*, digital serration*, max frontonasal fuse*, mand level frontonasal*, lower lid
138		V12j	47	hindlimb digital plate	**E**	digital grooves*, digital serration*, mand level frontonasal*
140		V14a	61	hatch	**L**	first claw, mand occlusion point
143		G01f	67	max frontonasal fuse	**E**	mand level frontonasal
144		G02c	70	mand posterior lens	**L**	forelimb ridge*, max bud*, max midline eye*
148		G03d	79	5th arch	**L**	11–15 somite pairs, 16–20 somite pairs*, forelimb ridge*, max bud*
152		T01a	88	cervical flexure 90°	**L**	olfactory pit, 3rd slit
153		T01b	89	cervical flexure disap-peared	**L**	finger*, mand posterior lens*, hyoid flap*
155		A01d	94	eyelid ventral lens	**L**	mand occlusion point, eyelid at scleral papillae
156		A01e	95	eyelid half eye	**L**	first claw, wrinkles on neck
160	Sauropsida (Node 40) → Sauria (Node 28)	V04e	11	16–20 somite pairs	**E**	otic pit, lens vesicle
164		V04i	15	36–40 somite pairs	**E**	forelimb elongated, forelimb AER, hindlimb paddle
180		G04d	84	4th slit	**L**	3rd slit
186	Sauria (Node 28) → Archosauria (Node 26)	V04a	7	somites hard count	**E**	forelimb elongated, forelimb AER, hindlimb paddle
201		V12e	42	hindlimb AER	**E**	forelimb elongated, forelimb AER
202		V12n	51	first claw	**L**	head scales, limb scales
203		G02d	71	mand midline eye	**L**	forelimb digital plate, hindlimb digital plate, max frontonasal fuse, slits closed
210		A01b	92	eyelid begun overgrow	**L**	head scales, mand occlusion point
286	Sauria (Node 28) → Lepidosauria (Node 27)		0	TWINS		Twins (21–25 somite pairs, lens vesicle) (whole forelimb scales, eyelid ventral lens)
294		V08d	29	contour lens/iris	**E**	max midline eye
297		V11	37	tail bud	**E**	mand arch bud
300		V13h	59	tail scales	**E**	head scales, neck scales
306		G03c	78	4th arch	**L**	max bud, 3rd slit
308		G04a	81	1st slit	**E**	anterior cephalic projection, 2nd slit
398	Sauropsida (Node 40) → Testudines (Node 39)	V05c	20	head projection disap-peared	**E**	throat scales, whole forelimb scales
401		V08e	30	pupil forms	**E**	hindlimb paddle
404		V13h	59	tail scales	**L**	first claw, throat scales, limb scales, whole forelimb scales
410		T01a	88	cervical flexure 90°	**L**	forelimb AER
411		T01b	89	cervical flexure disap-peared	**L**	first claw, whole forelimb scales, eyelid begun overgrow, eyelid half eye
412		A01a	91	lower lid	**L**	mand level frontonasal, caruncle
416	Testudines (Node 39) → Cryptodira (Node 38)	V06b	22	external nares	**L**	mand posterior eye
417		V07c	25	otic capsule inconspicu-ous	**E**	somites hard count, mand anterior lens
418		V08f	31	scleral papillae	**E**	carapace scales
426		G02c	70	mand posterior lens	**L**	somites hard count, forelimb elbow, hindlimb paddle, hindlimb digital plate
427		G03e	80	hyoid flap	**E**	forelimb AER, hindlimb AER
429		G04d	84	4th slit	**L**	max midline eye
438	Cryptodira (Node 38) → Chelonioidea (Node 30)	V04i	15	36–40 somite pairs	**E**	31–35 somite pairs, forelimb bud, forelimb elongated, hindlimb paddle

441		V07a	23	otic pit	**E**	ventricle S-shaped, mand arch bud
442		V07b	24	otic vesicle	**E**	ventricle S-shaped, mand arch bud
443		V07c	25	otic capsule inconspicu-ous	**E**	hindlimb paddle
446		V08d	29	contour lens/iris	**E**	tail bud, 4th arch
449		V12c	40	forelimb elongated	**E**	hindlimb AER
453		V13d	55	neck scales	**L**	head projection disappeared, throat scales, tail scales
462		A01d	94	eyelid ventral lens	**L**	head scales, throat scales, whole forelimb scales
463		A01f	96	membrana nictitans	**L**	throat scales, tail scales
465		S02c	101	carapace not anterior	**L**	forelimb elbow, digital grooves, urogenital papilla bud
466		S02d	102	carapace clearly limited	**L**	digital grooves, digital serration
468	Chelonioidea (Node 30) → Euchelonioidea (Node 29)	V03b	4	neural folds closure	**L**	6–10 somite pairs, head bulbus, anterior cephalic projection, otic pit, ventricle S–shaped
469		V03c	5	anterior neuropore closed	**L**	6–10 somite pairs, 11–15 somite pairs, otic pit
473		V05b	19	anterior cephalic projec-tion	**L**	otic pit, ventricle S-shaped
475		V08a	26	optic vesicle	**E**	1–5 somite pairs, otic pit
480		V10a	34	ventricle bulbus	**E**	otic pit, optic vesicle
483		V12f	43	forelimb elbow	**E**	somites hard count, pupil forms, hindlimb AER, carapacial ridge
484		V12h	45	hindlimb paddle	**E**	hindlimb AER, forelimb elbow, carapacial ridge
485		V12i	46	forelimb digital plate	**E**	somites hard count, hindlimb paddle, carapacial ridge
486		V12j	47	hindlimb digital plate	**E**	somites hard count, carapacial ridge
488		V13c	54	eyelid scales	**E**	throat scales, neck scales, whole forelimb scales, tail scales
489		V13f	57	limb scales	**E**	throat scales, tail scales
494		G02b	69	mand posterior eye	**L**	max bud, 3rd slit
495		G02d	71	mand midline eye	**L**	somites hard count, max frontonasal fuse
497		G03b	77	3rd arch	**L**	optic fissure, forelimb ridge
501		G04c	83	3rd slit	**L**	26–30 somite pairs
503		G05a	86	urogenital papilla bud	**E**	forelimb elongated, hindlimb AER
504		G05b	87	urogenital papilla inconspicuous	**L**	throat scales, whole forelimb scales, tail scales
505		T01a	88	cervical flexure 90°	**L**	hatch, membrana nictitans
509		A01b	92	eyelid begun overgrow	**E**	carapace scales, mand anterior lens
766	Cryptodira (Node 38) → Cryptodira/excl.Chelonioidea (Node 37)	V06b	22	external nares	**L**	forelimb elongated
774		G01f	67	max frontonasal fuse	**E**	mand anterior lens
775		G02a	68	mand arch bud	**E**	6–10 somite pairs
776		G02b	69	mand posterior eye	**L**	max bud, 4th arch
788		S02d	102	carapace clearly limited	**E**	thoracal bulbus disappeared, mand anterior lens
835	Cryptodira/excl.Chelonioidea (Node 37) → Trionychia/Testudinoidea (Node 36)	V07c	25	otic capsule inconspicu-ous	**E**	forelimb bud
836		V08d	29	contour lens/iris	**L**	max anterior eye
838		V10a	34	ventricle bulbus	**E**	anterior cephalic projection
840		V11	37	tail bud	**E**	2nd slit
845		G03e	80	hyoid flap	**L**	pupil forms, max anterior eye, max frontonasal fuse
862	Trionychia/Testudinoidea (Node 36) → Testudinoidea (Node 33)	V13f	57	limb scales	**E**	throat scales, wrinkles on neck, eyelid ventral lens
863		V13i	60	carapace scales	**E**	rhamphothecae
865		G01b	63	max posterior eye	**E**	4th arch, 2nd slit, 3rd slit
874		G04e	85	slits closed	**E**	forelimb elbow, forelimb digital plate, hindlimb digital plate
876		T01b	89	cervical flexure disap-peared	**E**	throat scales, eyelid half eye
891	Testudinoidea (Node 33) → Emydidae (Node 32)	V08f	31	scleral papillae	**L**	caruncle, carapace clearly limited
895		V13d	55	neck scales	**L**	throat scales
896		V13i	60	carapace scales	**E**	digital serration
907		T01b	89	cervical flexure disap-peared	**E**	head scales
918	Emydidae (Node 32) → *Chysemys picta*/*Trachemys scripta* (Node 31)	V12f	43	forelimb elbow	**L**	forelimb digital plate
1077	Trionychia/Testudinoidea (Node 36) → Trionychia (Node 35)	V06b	22	external nares	**L**	forelimb elbow, forelimb digital plate, hindlimb digital plate, digital grooves, mand posterior lens, mand anterior lens, urogenital papilla bud, lower lid, eyelid begun over-grow, carapacial ridge, longitudinal carapacial ridge, carapace not anterior, carapace clearly limited
1085		V12f	43	forelimb elbow	**L**	hindlimb digital plate
1087		V12m	50	finger	**L**	carapace scales
1097		G03e	80	hyoid flap	**L**	hindlimb digital plate, carapacial ridge
1105		S02f	104	carapace irregular	**L**	digital serration, mand anterior eye, mand level frontonasal
1115	Trionychia (Node 35) → Trionychinae (Node 34)	G02e	72	mand anterior lens	**E**	digital grooves, carapace clearly limited

Table S9 – Temporal shifts for species (unordered characters)

List of temporal shifts that are autapomorphic for the terminal branches (species) in a topology assuming the best-supported hypothesis (Sauria + Testudines/Cryptodira“b” relationship, Figure 5). First character occurs L=late or E=early in relation to the second character(s). For referred nodes compare Figure S3. For abbreviations, see Table S2.

shift no.	branch	standard event code …	character no. …	character …	… moved LATE (L) / EARLY (E) in relation to …	… characters (CONSENSUS)
24	Mammalia (Node 25) → *Tachyglossus aculeatus*	V03c	5	anterior neuropore closed	**L**	11–15 somite pairs, external nares, lens vesicle, ventricle bulbus, tail bud, forelimb elongated, forelimb paddle, hindlimb paddle, max midline eye, mand posterior eye, 3rd arch, 4th arch
28		V07a	23	otic pit	**L**	6–10 somite pairs, 11–15 somite pairs
31		V08a	26	optic vesicle	**L**	posterior neuropore closed, 6–10 somite pairs, 11–15 somite pairs, otic pit, ventricle bulbus, tail bud, 3rd arch, 4th arch
32		V08b	27	lens vesicle	**L**	external nares, forelimb elongated, forelimb paddle, hindlimb paddle
33		V08c	28	optic fissure	**E**	forelimb bud, max bud
34		V08d	29	contour lens/iris	**L**	max frontonasal fuse
35		V10a	34	ventricle bulbus	**L**	3rd arch, 4th arch, 3rd slit
38		V12b	39	forelimb bud	**E**	olfactory pit, max bud, max posterior eye, max midline eye, max anterior lens
41		G02b	69	mand posterior eye	**L**	external nares, forelimb elongated, forelimb paddle, hindlimb paddle, max midline eye
42		G02d	71	mand midline eye	**L**	somites hard count, hindlimb paddle, forelimb digital plate
46		G05a	86	Urogenital papilla bud	**L**	somites hard count, contour lens/iris, forelimb paddle, hindlimb paddle, forelimb digital plate, max frontonasal fuse, mand midline eye, mand anterior eye, lower lid
68	Theria (Node 24) → *Didelphis virginiana*	V03b	4	neural folds closure	**L**	head bulbus, otic pit, optic vesicle
69		V03c	5	anterior neuropore closed	**E**	posterior neuropore closed, head bulbus, otic pit, optic vesicle
73		V07b	24	otic vesicle	**L**	max posterior eye, max midline eye, cervical flexure 90°
84		G01f	67	max frontonasal fuse	**E**	lower lid
89		G02g	75	mand occlusion point	**L**	head projection disappeared, first claw, eyelid hald eye
94		T01b	89	cervical flexure disappeared	**L**	head projection disappeared, contour lens/iris, finger, mand occlusion point, eyelid hald eye
102	Theria (Node 24) → *Dasypus hybridus*	V07b	24	otic vesicle	**E**	ventricle bulbus, tail bud, mand arch bud
106		V10b	35	thoracal bulbus disappeared	**L**	head projection disappeared, first claw, eyelid hald eye
107		V12a	38	forelimb ridge	**L**	max bud, max posterior eye, max midline eye, 4th arch, cervical flexure 90°
108		V12i	46	forelimb digital plate	**E**	somites hard count
110		G01e	66	max anterior eye	**E**	forelimb paddle
111		G02d	71	mand midline eye	**E**	forelimb elongated, max bud, max posterior eye, max midline eye, max anterior lens, cervical flexure 90°
113		G02f	73	mand anterior eye	**E**	forelimb elongated, forelimb paddle, max anterior lens
114		G02g	74	mand level frontonasal	**E**	somites hard count, forelimb elongated, forelimb paddle, max anterior eye
115		G03a	76	2nd arch	**L**	tail bud, max posterior eye
117		G03c	78	4th arch	**L**	max bud, max posterior eye, max midline eye, 3rd arch, cervical flexure 90°
213	Archosauria (Node 26) → *Gallus gallus*	V03d	6	posterior neuropore closed	**L**	16–20 somite pairs, 21–25 somite pairs, anterior cephalic projection, tail bud, mand arch bud, 2nd arch
214		V05c	20	head projection disappeared	**E**	first claw, eyelid ventral lens
215		V07b	24	otic vesicle	**E**	anterior cephalic projection, 1st slit
216		V08b	27	lens vesicle	**E**	2nd arch, 1st slit
217		V08d	29	contour lens/iris	**L**	optic fissure, forelimb paddle, max midline eye, max anterior lens
218		V08e	30	pupil forms	**L**	back scales, max frontonasal fuse, mand anterior eye, caruncle
222		V12b	39	forelimb bud	**L**	forelimb AER, max bud, max midline eye
223		V12c	40	forelimb elongated	**L**	forelimb AER, max anterior lens
225		V12i	46	forelimb digital plate	**E**	forelimb elbow, hindlimb digital plate, max anterior lens
227		V12k	48	digital grooves	**E**	forelimb elbow, hindlimb digital plate
230		V13a	52	head scales	**E**	finger, back scales, mand occlusion point
231		V13b	53	throat scales	**E**	thoracal bulbus disappeared, finger, neck scales, back scales, tail scales, caruncle
232		V13d	55	neck scales	**E**	finger, back scales, mand occlusion point, caruncle
233		V13f	57	limb scales	**E**	finger, neck scales, back scales, tail scales, caruncle
236		G02a	68	mand arch bud	**L**	16–20 somite pairs, 2nd arch
243		T01b	89	cervical flexure disappeared	**E**	first claw
245	Archosauria (Node 26) → *Alligator mississippiensis*	V01a	1	egg lay	**L**	primitive streak, neural folds closure, 6–10 somite pairs, 11–15 somite pairs, head bulbus, optic vesicle
252		V08a	26	optic vesicle	**L**	head bulbus
253		V08b	27	lens vesicle	**L**	forelimb ridge, forelimb bud, max bud, 3rd slit
266		V13b	53	throat scales	**L**	limb scales, cervical flexure disappeared
267		V13h	59	tail scales	**L**	finger
274		G03b	77	3rd arch	**L**	forelimb bud, max bud
284		A01f	96	Membrane nictitans	**E**	head scales, mand occlusion point
314	Lepidosauria (Node 27) → *Sphenodon punctatus*	V03c	5	anterior neuropore closed	**L**	anterior cephalic projection, otic vesicle, ventricle bulbus, 1st slit
319		V05b	19	anterior cephalic projection	**L**	otic vesicle

320		V05c	20	head projection disappeared	**E**	scales, throat scales, eyelid scales, limb scales, whole forelimb scales, eyelid ventral lens
321		V06b	22	external nares	**L**	forelimb bud, max bud
323		V08f	31	scleral papillae	**E**	digital serration
325		V10b	35	thoracal bulbus disappeared	**E**	scleral papillae, digital serration, tail scales
327		V11	37	tail bud	**L**	3rd slit
329		V12m	50	Finger	**L**	throat scales, limb scales
336		V13h	59	tail scales	**E**	scleral papillae, digital serration
342		G02b	69	mand posterior eye	**L**	forelimb bud, max bud, 3rd slit
344		G03a	76	2nd arch	**L**	external nares, optic fissure, forelimb bud, max bud, max posterior eye, mand posterior eye, 3rd arch, 1st slit, 3rd slit
345		G03b	77	3rd arch	**L**	optic fissure, forelimb bud, max bud, max posterior eye, mand posterior eye, 3rd slit
348		G04b	82	2nd slit	**L**	21–optic fissure, 3rd slit
350		T01a	88	cervical flexure 90°	**L**	external nares, optic fissure, forelimb bud, max bud, max posterior eye
351		T01c	90	wrinkles on neck	**L**	head scales, throat scales, neck scales, limb scales
354		A02a	97	Caruncle	**L**	finger, head scales, throat scales, neck scales, back scales, limb scales, wrinkles on neck, eyelid begun overgrow
362	Lepidosauria (Node 27) → *Lacerta vivipara*	V06b	22	external nares	**E**	lens vesicle, max posterior eye
363		V07a	23	otic pit	**L**	11–15 somite pairs, optic vesicle, ventricle bulbus
370		V11	37	tail bud	**E**	11–15 somite pairs, ventricle bulbus, 1st slit
388		G04e	85	slits closed	**L**	digital serration, caruncle
389		T01a	88	cervical flexure 90°	**E**	11–15 somite pairs, lens vesicle, mand posterior eye
391		T01c	90	wrinkles on neck	**E**	eyelid begun overgrow
517	Euchelonioidea (Node 29) → *Caretta caretta*	V06a	21	olfactory pit	**E**	21–25 somite pairs, 26–30 somite pairs, optic fissure, contour lens/iris, forelimb ridge
518		V06b	22	external nares	**E**	36–40 somite pairs, 41–45 somite pairs, otic capsule inconspicuous, hindlimb paddle, mand posterior eye
519		V07b	24	otic vesicle	**L**	anterior neuropore closed, 11–15 somite pairs, ventricle S-shaped
521		V08a	26	optic vesicle	**L**	neural folds closure, 1–5 somite pairs, head bulbus, anterior cephalic projection, ventricle S-shaped
522		V08b	27	lens vesicle	**L**	16–20 somite pairs, optic fissure, forelimb ridge, 3rd arch, 1st slit
523		V10a	34	ventricle bulbus	**L**	neural folds closure, 1–5 somite pairs, 6–10 somite pairs, head bulbus, anterior cephalic projection, optic vesicle, ventricle S-shaped
525		V11	37	tail bud	**E**	anterior neuropore closed, 11–15 somite pairs, 16–20 somite pairs, contour lens/iris
529		V12l	49	digital serration	**E**	scleral papillae, digital grooves, caruncle
536		G01d	65	max anterior lens	**L**	41–45 somite pairs, forelimb paddle
537		G01e	66	max anterior eye	**E**	forelimb AER, hindlimb AER, forelimb elbow
538		G01f	67	max frontonasal fuse	**E**	somites hard count, carapacial ridge
539		G02a	68	mand arch bud	**L**	11–15 somite pairs, 16–20 somite pairs, otic vesicle, lens vesicle, optic fissure, ventricle S-shaped, forelimb ridge, 3rd arch, 1st slit
540		G02e	72	mand anterior lens	**L**	thoracal bulbus disappeared, lower lid, carapace clearly limited, carapace beyond tail
543		G03a	76	2nd arch	**L**	16–20 somite pairs, otic vesicle, lens vesicle, optic fissure, forelimb ridge, 1st slit
544		G03c	78	4th arch	**L**	26–30 somite pairs, 31–35 somite pairs, optic fissure
545		G03e	80	hyoid flap	**L**	41–45 somite pairs, forelimb AER, hindlimb AER, forelimb elbow, forelimb paddle
546		G04a	81	1st slit	**L**	optic fissure, forelimb ridge
547		G04b	82	2nd slit	**L**	41–45 somite pairs, optic fissure, forelimb ridge, forelimb elongated, forelimb paddle, max bud, max anterior lens, mand posterior eye, 4th arch, 3rd slit, 4th slit, urogenital papilla bud
548		G04c	83	3rd slit	**L**	26–30 somite pairs, 31–35 somite pairs, 36–40 somite pairs, 41–45 somite pairs, forelimb elongated, forelimb paddle, max bud, max anterior lens, mand posterior eye, 4th arch, urogenital papilla bud
549		G04d	84	4th slit	**L**	26–30 somite pairs, 31–35 somite pairs
550		G04e	85	slits closed	**L**	41–45 somite pairs, forelimb elongated, forelimb paddle, max anterior lens, mand posterior eye, urogenital papilla bud
551		G05a	86	urogenital papilla bud	**L**	41–45 somite pairs, mand posterior eye
552		T01a	88	cervical flexure 90°	**L**	somites hard count, forelimb elongated, hindlimb AER, forelimb elbow, forelimb paddle, urogenital papilla bud, carapacial ridge
553		T01c	90	wrinkles on neck	**E**	scleral papillae, caruncle
557		S02b	100	longitudinal carapacial ridge	**L**	slits closed
560		S02e	103	carapace beyond tail	**L**	carapace scales, eyelid begun overgrow
563	Euchelonioidea (Node 29) → *Chelonia mydas*	V05c	20	head projection disappeared	**L**	throat scales, neck scales, whole forelimb scales, tail scales, hatch, cervical flexure disappeared, eyelid at scleral papillae, eyelid ventral lens, eyelid hald eye, membrana nictitans
564		V06b	22	external nares	**E**	21–25 somite pairs, 26–30 somite pairs, 31–35 somite pairs, 36–40 somite pairs, 41–45 somite pairs, contour lens/iris, max bud, 2nd slit, 3rd slit, 4th slit, cervical flexure 90°
565		V07c	25	otic capsule inconspicuous	**L**	31–35 somite pairs, hindlimb AER, forelimb elbow, hyoid flap
568		V08c	28	optic fissure	**L**	26–30 somite pairs, contour lens/iris, 2nd slit, 3rd slit
569		V08e	30	pupil forms	**L**	somites hard count, otic capsule inconspicuous, carapacial ridge
570		V08f	31	scleral papillae	**E**	somites hard count, 41–45 somite pairs, hindlimb AER, forelimb elbow, forelimb paddle, lower lid, caruncle, carapacial ridge, carapace not anterior, carapace beyond tail
574		V12a	38	forelimb ridge	**L**	26–30 somite pairs, contour lens/iris, 2nd slit, 3rd slit
575		V12c	40	forelimb elongated	**L**	hyoid flap
576		V12d	41	forelimb AER	**E**	31–35 somite pairs, 36–40 somite pairs, 41–45 somite pairs, hindlimb AER, forelimb elbow, forelimb paddle, urogenital papilla bud
577		V12i	46	forelimb digital plate	**L**	somites hard count, forelimb elbow, hyoid flap, carapacial ridge
578		V12j	47	hindlimb digital plate	**L**	somites hard count, hyoid flap, carapacial ridge
581		V12n	51	first claw	**L**	eyelid at scleral papillae
582		V13a	52	head scales	**E**	throat scales, neck scales, tail scales
583		V13f	57	limb scales	**L**	eyelid at scleral papillae
584		V13g	58	whole forelimb scales	**L**	throat scales, tail scales
585		G01a	62	max bud	**E**	21–25 somite pairs, contour lens/iris, 2nd slit, 3rd slit
586		G01d	65	max anterior lens	**E**	26–30 somite pairs, 31–35 somite pairs, 36–40 somite pairs, 3rd slit, 4th slit, urogenital papilla bud
587		G01e	66	max anterior eye	**E**	41–45 somite pairs, hindlimb AER, forelimb elbow, forelimb paddle

588		G01f	67	max frontonasal fuse	**L**	rib primordia, slits closed
592		G02d	71	mand midline eye	**L**	digital serration, carapace scales, eyelid begun overgrow
593		G02f	73	mand anterior eye	**L**	thoracal bulbus disappeared, carapace scales, wrinkles on neck, eyelid at scleral papillae
595		G02g	75	mand occlusion point	**L**	throat scales, neck scales, tail scales, eyelid at scleral papillae
597		G03b	77	3rd arch	**L**	26–30 somite pairs, contour lens/iris, 4th arch, 2nd slit, 3rd slit
599		G03e	80	hyoid flap	**L**	41–45 somite pairs, forelimb paddle
600		G04e	85	slits closed	**L**	digital grooves
602		T01a	88	cervical flexure 90°	**E**	31–35 somite pairs, 36–40 somite pairs, forelimb AER
603		T01c	90	wrinkles on neck	**L**	first claw, limb scales, carapace scales, eyelid at scleral papillae
605		S01a	98	rhamphothecae	**L**	thoracal bulbus disappeared, first claw, throat scales, neck scales, limb scales, tail scales, carapace scales, mand level frontonasal, mand occlusion point, wrinkles on neck, eyelid begun overgrow, eyelid at scleral papillae
606		S02d	102	carapace clearly limited	**E**	carapace scales, lower lid
607	Euchelonioidea (Node 29) → *Eretmochelys imbricata*	V05a	18	head bulbus	**L**	otic pit, optic vesicle
608		V06b	22	external nares	**L**	rib primordia, forelimb AER, hindlimb AER, digital grooves
610		V07c	25	otic capsule incon-spicuous	**E**	max anterior lens, urogenital papilla bud
614		V11	37	tail bud	**L**	optic fissure, forelimb ridge
615		V12i	46	forelimb digital plate	**E**	31–35 somite pairs, 36–40 somite pairs, 41–45 somite pairs, forelimb paddle, max anterior lens, mand posterior eye
616		V12j	47	hindlimb digital plate	**E**	31–35 somite pairs, 36–40 somite pairs, 41–45 somite pairs, otic capsule inconspicuous
619		V13c	54	eyelid scales	**E**	head scales
623		G01d	65	max anterior lens	**E**	36–40 somite pairs
624		G01e	66	max anterior eye	**L**	somites hard count, rib primordia, digital grooves, mand midline eye, carapacial ridge, carapace not anterior
626		G02e	72	mand anterior lens	**L**	digital serration, carapace clearly limited
627		G02g	74	mand level frontona-sal	**L**	head projection disappeared, head scales, mand occlusion point
628		G02g	75	mand occlusion point	**L**	head projection disappeared, eyelid at scleral papillae
630		G03e	80	hyoid flap	**E**	36–40 somite pairs, max anterior lens, mand posterior eye, urogenital papilla bud
631		G04e	85	slits closed	**E**	41–45 somite pairs, forelimb AER, hindlimb AER, forelimb paddle
634		T01a	88	cervical flexure 90°	**L**	somites hard count, hindlimb AER, forelimb elbow, forelimb paddle, hindlimb paddle, carapacial ridge
636		A01a	91	lower lid	**E**	rib primordia, digital grooves, caruncle
637		S01a	98	rhamphothecae	**E**	rib primordia, digital grooves
640	Euchelonioidea (Node 29) → *Lepidochelys olivacea*	V03b	4	neural folds closure	**E**	1–5 somite pairs, 6–10 somite pairs, head bulbus, ventricle S–shaped
641		V03c	5	anterior neuropore closed	**E**	6–10 somite pairs, 11–15 somite pairs, head bulbus, ventricle S–shaped
644		V04d	10	11–15 somite pairs	**E**	ventricle S–shaped
646		V05c	20	head projection disappeared	**E**	first claw, head scales, eyelid scales, mand level frontonasal, mand occlusion point
647		V06b	22	external nares	**L**	rib primordia, forelimb elongated, max anterior eye, max frontonasal fuse, mand posterior lens, lower lid, caruncle, longitudinal carapacial ridge, carapace not anterior
648		V07b	24	otic vesicle	**L**	lens vesicle, 1st slit
649		V07c	25	otic capsule incon-spicuous	**L**	somites hard count, 31–35 somite pairs, forelimb paddle, hindlimb paddle, max anterior eye, mand posterior eye, carapacial ridge
650		V08d	29	contour lens/iris	**L**	26–30 somite pairs, 31–35 somite pairs, forelimb ridge, forelimb bud
653		V10b	35	thoracal bulbus disappeared	**E**	caruncle, carapace beyond tail
654		V11	37	tail bud	**E**	posterior neuropore closed, 16–20 somite pairs, lens vesicle, 2nd slit
657		V12h	45	hindlimb paddle	**L**	forelimb paddle, max anterior eye
658		V12k	48	digital grooves	**E**	somites hard count, pupil forms, max anterior eye, carapacial ridge, longitudinal carapacial ridge
659		V13d	55	neck scales	**E**	first claw, head scales, eyelid scales, mand level frontonasal, mand occlusion point
663		G01d	65	max anterior lens	**L**	forelimb paddle, cervical flexure 90°
665		G02a	68	mand arch bud	**L**	26–30 somite pairs, 31–35 somite pairs, otic vesicle, lens vesicle, contour lens/iris, forelimb ridge, forelimb bud, max midline eye, 1st slit
666		G02b	69	mand posterior eye	**L**	31–35 somite pairs, forelimb bud, forelimb elongated, forelimb paddle, hindlimb paddle, max midline eye, max anterior lens, max anterior eye, urogenital papilla bud, cervical flexure 90°
668		G02f	73	mand anterior eye	**E**	caruncle, carapace beyond tail
670		G03a	76	2nd arch	**L**	26–30 somite pairs, 31–35 somite pairs, otic vesicle, lens vesicle, contour lens/iris, forelimb ridge, forelimb bud, max midline eye, 1st slit
672		G04e	85	slits closed	**E**	26–30 somite pairs, 31–35 somite pairs, 36–40 somite pairs, 41–45 somite pairs, pupil forms, forelimb bud, forelimb paddle, max bud, longitudinal carapacial ridge
674		T01a	88	cervical flexure 90°	**L**	forelimb elongated, forelimb paddle
675		T01b	89	cervical flexure disappeared	**E**	head projection disappeared, thoracal bulbus disappeared, first claw, head scales, eyelid scales, neck scales, hatch, mand anterior lens, mand anterior eye, mand level frontonasal, mand occlusion point, caruncle, longitudinal carapacial ridge, carapace beyond tail, carapace irregular
676		T01c	90	wrinkles on neck	**L**	first claw, mand occlusion point
678		S02d	102	carapace clearly limited	**L**	first claw, mand occlusion point, carapace irregular
680	Euchelonioidea (Node 29) → *Natator depressus*	V06b	22	external nares	**L**	rib primordia, forelimb AER, hindlimb AER, digital grooves
682		V07c	25	otic capsule incon-spicuous	**E**	max anterior lens, urogenital papilla bud
685		V11	37	tail bud	**L**	optic fissure, forelimb ridge
686		V12i	46	forelimb digital plate	**E**	31–35 somite pairs, 36–40 somite pairs, 41–45 somite pairs, forelimb paddle, max anterior lens, mand posterior eye
687		V12j	47	hindlimb digital plate	**E**	31–35 somite pairs, 36–40 somite pairs, 41–45 somite pairs, otic capsule inconspicuous, forelimb paddle, max anterior lens, mand posterior eye, urogenital papilla bud
693		G01d	65	max anterior lens	**E**	36–40 somite pairs
694		G01e	66	max anterior eye	**L**	somites hard count, rib primordia, thoracal bulbus disappeared, digital grooves, caruncle, carapacial ridge, carapace beyond tail
697		G02f	73	mand anterior eye	**E**	somites hard count, 41–45 somite pairs, rib primordia, forelimb AER, hindlimb AER, forelimb elbow, forelimb paddle, hindlimb paddle, digital grooves, slits closed, carapacial ridge, carapace not anterior
699		G03e	80	hyoid flap	**E**	36–40 somite pairs, max anterior lens, mand posterior eye, urogenital papilla bud
700		G04e	85	slits closed	**E**	41–45 somite pairs, forelimb AER, hindlimb AER, forelimb paddle

702		T01a	88	cervical flexure 90°	**E**	26–30 somite pairs, 31–35 somite pairs, 36–40 somite pairs, olfactory pit, forelimb AER, max anterior lens, mand posterior eye, 3rd slit, 4th slit
703		T01c	90	wrinkles on neck	**E**	thoracal bulbus disappeared, digital serration
704		A01a	91	lower lid	**E**	rib primordia, digital grooves, carapace not anterior
705		A01f	96	membrana nictitans	**E**	throat scales, whole forelimb scales, tail scales
707		S01a	98	rhamphothecae	**E**	rib primordia, digital grooves, digital serration
708		S02c	101	carapace not anterior	**E**	rib primordia, digital grooves
709		S02f	104	carapace irregular	**L**	first claw, mand occlusion point
710	Chelonioidea (Node 30) → *Dermochelys coriacea*		0	TWINS		Twins (1–5 somite pairs, primitive streak)
711		V03c	5	anterior neuropore closed	**E**	anterior cephalic projection, ventricle S–shaped
712		V04a	7	somites hard count	**E**	forelimb AER, hindlimb AER, max anterior eye
713		V04c	9	6–10 somite pairs	**L**	otic pit, ventricle S–shaped
716		V05c	20	head projection disappeared	**L**	throat scales, tail scales
717		V06a	21	olfactory pit	**L**	max bud
718		V06b	22	external nares	**E**	somites hard count, 31–35 somite pairs, 36–40 somite pairs, forelimb bud, cervical flexure 90°
719		V07c	25	otic capsule incon-spicuous	**E**	31–35 somite pairs, pupil forms, forelimb bud, forelimb elongated, 4th slit, cervical flexure 90°
720		V08b	27	lens vesicle	**E**	ventricle S–shaped
721		V08d	29	contour lens/iris	**E**	16–20 somite pairs, 21–25 somite pairs, optic fissure, forelimb ridge, 2nd slit
722		V08e	30	pupil forms	**E**	31–35 somite pairs, 36–40 somite pairs, 41–45 somite pairs, forelimb bud, forelimb elongated, forelimb AER, hindlimb AER, 4th slit, cervical flexure 90°
723		V08f	31	scleral papillae	**L**	head projection disappeared, eyelid scales
725		V10a	34	ventricle bulbus	**L**	6–10 somite pairs, otic vesicle, ventricle S-shaped, mand arch bud
726		V11	37	tail bud	**E**	16–20 somite pairs, 2nd slit
730		V12l	49	digital serration	**L**	rhamphothecae
733		V13f	57	limb scales	**L**	head scales
735		V13i	60	carapace scales	**L**	head projection disappeared, head scales, throat scales, eyelid scales, neck scales, tail scales
736		G01a	62	max bud	**L**	5th arch
740		G02b	69	mand posterior eye	**E**	optic fissure
741		G02d	71	mand midline eye	**E**	41–45 somite pairs, forelimb elongated, forelimb AER, hindlimb AER, max anterior eye
742		G02e	72	mand anterior lens	**E**	somites hard count, forelimb AER, hindlimb AER, forelimb elbow, forelimb digital plate, digital grooves, max anterior eye, carapacial ridge
743		G02g	74	mand level frontona-sal	**E**	caruncle, carapace clearly limited
744		G02g	75	mand occlusion point	**E**	thoracal bulbus disappeared, mand level frontonasal, caruncle, carapace clearly limited
746		G03b	77	3rd arch	**E**	16–20 somite pairs, tail bud
748		G04a	81	1st slit	**L**	optic fissure, forelimb ridge
749		G04c	83	3rd slit	**E**	21–25 somite pairs, 4th slit
750		G04e	85	slits closed	**L**	scleral papillae, digital serration, lower lid, carapace not anterio
751		G05a	86	urogenital papilla bud	**L**	forelimb elbow, hindlimb paddle, max anterior lens, carapacial ridge
752		T01a	88	cervical flexure 90°	**E**	31–35 somite pairs, forelimb AER
753		T01b	89	cervical flexure disappeared	**E**	head scales, throat scales, eyelid scales, whole forelimb scales, tail scales, urogenital papilla inconspicu-ous, wrinkles on neck, eyelid hald eye
755		A01b	92	eyelid begun over-grow	**L**	head projection disappeared, head scales, throat scales, eyelid scales, neck scales, limb scales, tail scales
756		A01c	93	eyelid at scleral papillae	**L**	head projection disappeared, throat scales, eyelid scales, neck scales, tail scales
790	Cryptodira/excl.Chelonioidea (Node 37) → *Chelydra serpentina*	V03b	4	neural folds closure	**E**	primitive streak, 1–5 somite pairs
791		V03c	5	anterior neuropore closed	**E**	anterior cephalic projection, optic vesicle
792		V03d	6	posterior neuropore closed	**E**	somite pairs, head bulbus, optic vesicle
793		V04a	7	somites hard count	**E**	forelimb elongated, forelimb AER, hindlimb AER, max anterior eye
794		V04c	9	6–10 somite pairs	**E**	anterior cephalic projection, optic vesicle
795		V04h	14	31–35 somite pairs	**E**	forelimb bud, max posterior eye
797		V05c	20	head projection disappeared	**E**	first claw, head scales
798		V06a	21	olfactory pit	**E**	4th arch, 2nd slit
800		V07c	25	otic capsule incon-spicuous	**L**	hindlimb digital plate
805		V12a	38	forelimb ridge	**E**	16–20 somite pairs, lens vesicle, 1st slit, 2nd
806		V12g	44	forelimb paddle	**L**	forelimb elongated, forelimb AER, hindlimb AER, forelimb digital plate, hindlimb digital plate, max frontonasal fuse, carapacial ridge
807		V12h	45	hindlimb paddle	**L**	otic capsule inconspicuous
808		V13b	53	throat scales	**E**	head projection disappeared, first claw, head scales, eyelid scales, whole forelimb scales
809		V13d	55	neck scales	**E**	finger, mand occlusion point, wrinkles on neck, eyelid begun overgrow
811		V13h	59	tail scales	**E**	head projection disappeared, finger, first claw, head scales, throat scales, limb scales, whole forelimb scales, mand occlusion point, wrinkles on neck, eyelid begun overgrow
812		G01a	62	max bud	**L**	forelimb bud, forelimb elongated, forelimb AER, hindlimb AER, max posterior eye, max midline eye, 5th arch
815		G02a	68	mand arch bud	**E**	1–5 somite pairs, head bulbus, anterior cephalic projection, optic vesicle
816		G02b	69	mand posterior eye	**L**	otic capsule inconspicuous, forelimb bud, max midline eye, longitudinal carapacial ridge
817		G02c	70	mand posterior lens	**L**	scleral papillae, digital grooves, carapace clearly limited
818		G02e	72	mand anterior lens	**L**	scleral papillae, digital serration
819		G02g	75	mand occlusion point	**E**	finger, eyelid begun overgrow
820		G03a	76	2nd arch	**E**	anterior neuropore closed, 11–15 somite pairs, anterior cephalic projection, otic pit, optic vesicle
822		G03e	80	hyoid flap	**E**	forelimb bud, forelimb elongated, max posterior eye
823		G04e	85	slits closed	**E**	somites hard count, contour lens/iris, pupil forms, forelimb elongated, forelimb AER, hindlimb AER, forelimb elbow, forelimb digital plate, hindlimb digital plate, max anterior eye
826		S01a	98	rhamphothecae	**L**	mand level frontonasal, eyelid begun overgrow

827		S02e	103	carapace beyond tail	**L**	finger, mand level frontonasal, wrinkles on neck, eyelid begun overgrow
828		S02f	104	carapace irregular	**E**	thoracal bulbus disappeared, caruncle
937	*Chysemys picta*/*Trachemys scripta* (Node 31) → *Chrysemys picta*	V06a	21	olfactory pit	**E**	forelimb elongated, 2nd arch, 2nd slit
938		V06b	22	external nares	**E**	hyoid flap
949		V13d	55	neck scales	**E**	wrinkles on neck
957		G03d	79	5th arch	**L**	forelimb elongated, forelimb paddle, 4th arch, 3rd slit, 4th slit
961		G04d	84	4th slit	**L**	3rd slit
962		G04e	85	slits closed	**E**	forelimb elongated, forelimb paddle, forelimb digital plate, hyoid flap
963		G05a	86	urogenital papilla bud	**L**	somites hard count, hindlimb digital plate, digital grooves, 4th slit, carapace clearly limited
965		T01a	88	cervical flexure 90°	**L**	somites hard count, contour lens/iris, forelimb elongated, forelimb paddle, hindlimb digital plate
968		A01b	92	eyelid begun overgrow	**L**	scleral papillae inconspicuous, first claw
971		S02f	104	carapace irregular	**E**	digital grooves, carapace clearly limited
972	*Chysemys picta*/*Trachemys scripta* (Node 31) → *Trachemys scripta*	V06b	22	external nares	**L**	scleral papillae, thoracal bulbus disappeared, forelimb elbow, forelimb paddle, forelimb digital plate, hindlimb digital plate, digital grooves, digital serration, finger, first claw, neck scales, slits closed, wrinkles on neck, carapace clearly limited, carapace beyond tail, carapace irregular
973		V07a	23	otic pit	**L**	olfactory pit, forelimb paddle, forelimb digital plate, hindlimb digital plate, digital grooves, 2nd arch, 3rd arch, hyoid flap, 1st slit, slits closed, cervical flexure 90°, carapace clearly limited
975		V12f	43	forelimb elbow	**L**	scleral papillae, hindlimb digital plate, digital grooves, digital serration, carapace clearly limited
979		V13h	59	tail scales	**E**	neck scales, whole forelimb scales, cervical flexure disappeared
980		V13i	60	carapace scales	**E**	digital grooves, carapacial ridge, carapace clearly limited
983		G02f	73	mand anterior eye	**E**	digital grooves, digital serration, carapacial ridge, carapace clearly limited
984		G02g	74	mand level frontonasal	**E**	digital grooves, digital serration, carapace clearly limited
985		G05a	86	urogenital papilla bud	**E**	forelimb elongated, 5th arch
989		S01a	98	rhamphothecae	**E**	digital grooves, digital serration, carapace clearly limited
991		S02e	103	carapace beyond tail	**E**	finger
992	Emydidae (Node 32) → *Graptemys nigrinoda*	V06b	22	external nares	**E**	forelimb ridge, max midline eye, mand posterior eye, cervical flexure 90°
993		V07b	24	otic vesicle	**L**	forelimb ridge, max midline eye, mand posterior eye
994		V07c	25	otic capsule inconspicuous	**L**	mand anterior lens, carapacial ridge, carapace clearly limited
995		V08b	27	lens vesicle	**L**	forelimb ridge, max midline eye, mand posterior eye
996		V08c	28	optic fissure	**L**	forelimb elongated, max midline eye, mand posterior eye, 4th slit
1005		V12n	51	first claw	**E**	wrinkles on neck
1013		G03a	76	2nd arch	**L**	otic vesicle, forelimb ridge, forelimb elongated, max midline eye, mand posterior eye
1014		G03c	78	4th arch	**L**	forelimb elongated
1015		G04a	81	1st slit	**L**	lens vesicle, forelimb ridge, forelimb elongated, max midline eye, mand posterior eye
1016		G04c	83	3rd slit	**L**	forelimb elongated, max midline eye, mand posterior eye
1019		G05a	86	urogenital papilla bud	**E**	forelimb elongated, forelimb AER, forelimb paddle, hindlimb paddle
1020		T01a	88	cervical flexure 90°	**E**	forelimb ridge, forelimb AER
1022		A01a	91	lower lid	**E**	carapace beyond tail
1025		A01d	94	eyelid ventral lens	**E**	wrinkles on neck
1026		A01e	95	eyelid hald eye	**E**	head scales, throat scales, eyelid scales, whole forelimb scales
1032	Testudinoidea (Node 33) → *Testudo hermanni*	V03d	6	posterior neuropore closed	**L**	anterior neuropore closed, 16–20 somite pairs, anterior cephalic projection, otic vesicle, lens vesicle, ventricle bulbus, tail bud, 1st slit
1036		V05c	20	head projection disappeared	**E**	scleral papillae inconspicuous, first claw, wrinkles on neck
1037		V06a	21	olfactory pit	**L**	forelimb bud, forelimb AER, max midline eye
1038		V06b	22	external nares	**L**	somites hard count, forelimb paddle
1039		V08b	27	lens vesicle	**L**	16–20 somite pairs
1040		V08c	28	optic fissure	**L**	forelimb bud, 3rd slit
1045		V12g	44	forelimb paddle	**L**	somites hard count
1046		V13a	52	head scales	**E**	scleral papillae inconspicuous, throat scales, limb scales, whole forelimb scales, tail scales
1047		V13d	55	neck scales	**E**	finger, carapace scales, mand occlusion point
1049		V13g	58	whole forelimb scales	**E**	first claw, throat scales
1050		G01a	62	max bud	**E**	anterior cephalic projection, mand arch bud, 3rd arch
1052		G01e	66	max anterior eye	**L**	somites hard count, external nares, contour lens/iris
1053		G01f	67	max frontonasal fuse	**L**	scleral papillae, forelimb digital plate, hindlimb digital plate, digital grooves, carapace clearly limited
1054		G03a	76	2nd arch	**E**	anterior cephalic projection, mand arch bud
1061		G05a	86	urogenital papilla bud	**L**	somites hard count, pupil forms, hindlimb digital plate
1125	Trionychinae (Node 34) → *Apalone spinifera*	V06a	21	olfactory pit	**L**	forelimb AER, max anterior eye
1126		V06b	22	external nares	**L**	scleral papillae inconspicuous, first claw, head scales, eyelid scales, neck scales, limb scales, eyelid hald eye, caruncle
1127		V08d	29	contour lens/iris	**L**	forelimb digital plate, hindlimb digital plate, max frontonasal fuse, carapacial ridge, longitudinal carapacial ridge
1128		V08e	30	pupil forms	**L**	forelimb elbow, forelimb digital plate, digital grooves, max frontonasal fuse, mand anterior lens, carapacial ridge, longitudinal carapacial ridge, carapace clearly limited
1130		V08g	32	scleral papillae inconspicuous	**L**	head scales, eyelid scales, neck scales, limb scales
1132		V12a	38	forelimb ridge	**L**	forelimb AER, 4th arch, 5th arch, 2nd slit
1133		V12f	43	forelimb elbow	**L**	digital grooves, mand anterior lens, longitudinal carapacial ridge, carapace clearly limited
1137		V13f	57	limb scales	**L**	head scales, eyelid scales, neck scales
1140		G02g	75	mand occlusion point	**E**	finger, carapace scales, carapace irregular
1141		G03a	76	2nd arch	**L**	forelimb AER, max posterior eye, 3rd arch, 4th arch, 5th arch
1149		S01a	98	rhamphothecae	**E**	scleral papillae, digital grooves, carapace clearly limited
1156	Trionychinae (Node 34) → *Pelodiscus sinensis*	V06a	21	olfactory pit	**E**	forelimb ridge, max posterior eye, 2nd arch
1157		V06b	22	external nares	**E**	digital grooves, mand anterior lens, carapace clearly limited
1160		V08d	29	contour lens/iris	**E**	olfactory pit, forelimb ridge, forelimb elongated, forelimb AER, max posterior eye, max anterior eye,

						2nd arch, 3rd arch, 5th arch
1161		V08e	30	pupil forms	**E**	somites hard count, forelimb elongated, max anterior eye, urogenital papilla bud
1162		V08f	31	scleral papillae	**L**	digital grooves, digital serration, caruncle, carapace clearly limited
1167		V12e	42	hindlimb AER	**L**	somites hard count, forelimb AER, max anterior eye, urogenital papilla bud
1168		V12f	43	forelimb elbow	**E**	somites hard count, forelimb elongated, forelimb digital plate, hindlimb digital plate, max anterior eye, urogenital papilla bud, carapacial ridge
1170		V12i	46	forelimb digital plate	**E**	somites hard count, forelimb elongated, hindlimb digital plate, max anterior eye, max frontonasal fuse, carapacial ridge
1180		G02c	70	mand posterior lens	**E**	hindlimb digital plate, max frontonasal fuse, carapacial ridge, longitudinal carapacial ridge
1183		G02g	74	mand level frontona-sal	**L**	scleral papillae inconspicuous, thoracal bulbus disappeared, finger, carapace scales, eyelid at scleral papillae
1184		G02g	75	mand occlusion point	**L**	scleral papillae inconspicuous, neck scales, limb scales, wrinkles on neck
1193		T01a	88	cervical flexure 90°	**E**	forelimb ridge, forelimb AER, max posterior eye, 2nd arch, 3rd arch, 5th arch, 1st slit
1194		T01b	89	cervical flexure disappeared	**E**	head scales, eyelid scales, neck scales, eyelid hald eye
1195		T01c	90	wrinkles on neck	**L**	scleral papillae inconspicuous, thoracal bulbus disappeared, first claw, head scales, eyelid scales, neck scales, limb scales
1196		A01a	91	lower lid	**E**	digital grooves, mand anterior lens, caruncle, longitudinal carapacial ridge, carapace clearly limited
1197		A01b	92	eyelid begun over-grow	**E**	digital grooves, mand anterior lens, caruncle, carapace clearly limited
1204		S02f	104	carapace irregular	**L**	scleral papillae inconspicuous, finger, first claw, neck scales, eyelid at scleral papillae
1206		V04a	7	somites hard count	**L**	pupil forms, forelimb elbow, forelimb digital plate, hindlimb digital plate, digital grooves, max frontona-sal fuse, mand posterior lens, carapacial ridge, longitudinal carapacial ridge, carapace not anterior, carapace clearly limited
1207		V04d	10	11–15 somite pairs	**L**	forelimb elongated, forelimb paddle, 2nd arch
1209		V05a	18	head bulbus	**E**	head scales, limb scales
1210		V06a	21	olfactory pit	**L**	somites hard count, contour lens/iris, pupil forms, forelimb elongated, forelimb elbow, forelimb paddle, hindlimb digital plate, digital grooves, carapace scales, max anterior eye, max frontonasal fuse, mand posterior eye, mand posterior lens, mand anterior lens, mand anterior eye, mand level frontonasal, hyoid flap, urogenital papilla bud, carapacial ridge, longitudinal carapacial ridge, carapace not anterior, carapace clearly limited, carapace irregular
1211		V06b	22	external nares	**L**	mand anterior eye, eyelid at scleral papillae
1212		V07a	23	otic pit	**L**	somites hard count, 11–15 somite pairs, optic fissure, contour lens/iris, forelimb elongated, forelimb elbow, forelimb paddle, hindlimb digital plate, digital grooves, mand anterior lens, 2nd arch, 3rd arch, hyoid flap, urogenital papilla bud, carapacial ridge, longitudinal carapacial ridge, carapace not anterior, carapace clearly limited
1213		V07b	24	otic vesicle	**L**	somites hard count, pupil forms, forelimb elongated, forelimb elbow, forelimb paddle, hindlimb digital plate, digital grooves, max anterior eye, max frontonasal fuse, mand anterior lens, mand anterior eye, mand level frontonasal, hyoid flap, urogenital papilla bud, carapacial ridge, longitudinal carapacial ridge, carapace not anterior, carapace clearly limited
1214	Trionychia (Node 35) → *Carettochelys insculpta*	V07c	25	otic capsule incon-spicuous	**L**	somites hard count, forelimb elbow, hindlimb digital plate, digital grooves, finger, limb scales, carapace scales, max frontonasal fuse, mand posterior lens, mand anterior lens, mand anterior eye, mand level frontonasal, mand occlusion point, hyoid flap, eyelid begun overgrow, eyelid at scleral papillae, cara-pacial ridge, longitudinal carapacial ridge, carapace not anterior, carapace clearly limited, carapace irregular
1215		V08c	28	optic fissure	**L**	pupil forms, forelimb elongated, forelimb paddle, hindlimb digital plate, max anterior eye, max frontona-sal fuse, hyoid flap, carapacial ridge
1216		V08d	29	contour lens/iris	**L**	pupil forms, hindlimb digital plate, max frontonasal fuse, carapacial ridge
1217		V08e	30	pupil forms	**L**	max frontonasal fuse, carapacial ridge
1218		V09	33	rib primordia	**E**	pupil forms, forelimb elbow, forelimb digital plate, hindlimb digital plate, digital grooves, max frontona-sal fuse, hyoid flap, carapacial ridge, longitudinal carapacial ridge, carapace clearly limited
1221		V12l	49	digital serration	**E**	pupil forms, scleral papillae, rib primordia, forelimb elbow, hindlimb digital plate, digital grooves, max anterior eye, max frontonasal fuse, hyoid flap, carapacial ridge, longitudinal carapacial ridge, carapace not anterior, carapace clearly limited
1223		V12n	51	first claw	**E**	eyelid begun overgrow, eyelid at scleral papillae
1224		V13c	54	eyelid scales	**E**	head scales, whole forelimb scales
1225		G02b	69	mand posterior eye	**L**	pupil forms, forelimb elbow, longitudinal carapacial ridge
1226		G02c	70	mand posterior lens	**L**	digital grooves, Carapace clearly limited
1229		G03a	76	2nd arch	**L**	forelimb elongated, forelimb paddle
1230		G03b	77	3rd arch	**L**	forelimb elongated, forelimb paddle, max anterior eye
1231		G05a	86	urogenital papilla bud	**L**	pupil forms, forelimb elbow, hindlimb digital plate, digital grooves, max frontonasal fuse, mand posterior lens, hyoid flap, carapacial ridge, longitudinal carapacial ridge, carapace clearly limited
1232		T01c	90	wrinkles on neck	**E**	eyelid begun overgrow, carapace irregular
1234	Testudines (Node 39) → *Emydura subglubosa*		0	TWINS		Twins (hatch, cervical flexure disappeared)
1236		V06b	22	external nares	**E**	31–35 somite pairs, optic fissure, max midline eye, 4th arch, 5th arch, 3rd slit, 4th slit
1237		V07a	23	otic pit	**L**	21–25 somite pairs, otic vesicle, optic fissure, forelimb ridge, 2nd arch, 3rd arch, 1st slit, 2nd slit
1238		V07b	24	otic vesicle	**L**	optic fissure, 2nd slit
1240		V08d	29	contour lens/iris	**E**	4th arch, 5th arch
1242		V08f	31	scleral papillae	**L**	finger, first claw, caruncle
1244		V12g	44	forelimb paddle	**E**	31–35 somite pairs, forelimb bud, hindlimb paddle, max anterior lens
1246		G01c	64	max midline eye	**E**	optic fissure, 5th arch, 3rd slit
1247		G01d	65	max anterior lens	**E**	forelimb bud
1248		G02b	69	mand posterior eye	**E**	optic fissure, contour lens/iris
1249		G02c	70	mand posterior lens	**E**	31–35 somite pairs, forelimb bud, forelimb paddle, urogenital papilla bud
1252		G02g	74	mand level frontona-sal	**E**	caruncle, carapace clearly limited, carapace beyond tail
1253		G02g	75	mand occlusion point	**E**	thoracal bulbus disappeared, finger, mand level frontonasal, eyelid begun overgrow, caruncle, carapace clearly limited, carapace beyond tail
1254		G03a	76	2nd arch	**L**	21–25 somite pairs, optic fissure, forelimb ridge
1255		G03b	77	3rd arch	**L**	21–25 somite pairs, forelimb ridge, 4th arch
1256		G03d	79	5th arch	**E**	optic fissure
1258		G04a	81	1st slit	**L**	optic fissure, forelimb ridge
1260		G04d	84	4th slit	**E**	mand posterior eye
1261		G04e	85	slits closed	**L**	lower lid, caruncle
1262		G05a	86	urogenital papilla bud	**E**	31–35 somite pairs, forelimb bud

1263		G05b	87	urogenital papilla inconspicuous	**L**	throat scales, tail scales
1264		T01a	88	cervical flexure 90°	**L**	somites hard count, 36–40 somite pairs, slits closed, lower lid, caruncle
1265		A01b	92	eyelid begun over-grow	**E**	thoracal bulbus disappeared, finger, mand level frontonasal, caruncle, carapace clearly limited, carapace beyond tail
1266		A01d	94	eyelid ventral lens	**E**	thoracal bulbus disappeared, finger, first claw, neck scales, tail scales, mand level frontonasal, mand occlusion point, eyelid begun overgrow, caruncle, carapace clearly limited, carapace beyond tail
1267		S01a	98	rhamphothecae	**L**	finger, first claw, carapace scales
1268		S02f	104	carapace irregular	**L**	finger, first claw, throat scales, tail scales, carapace scales, urogenital papilla inconspicuous

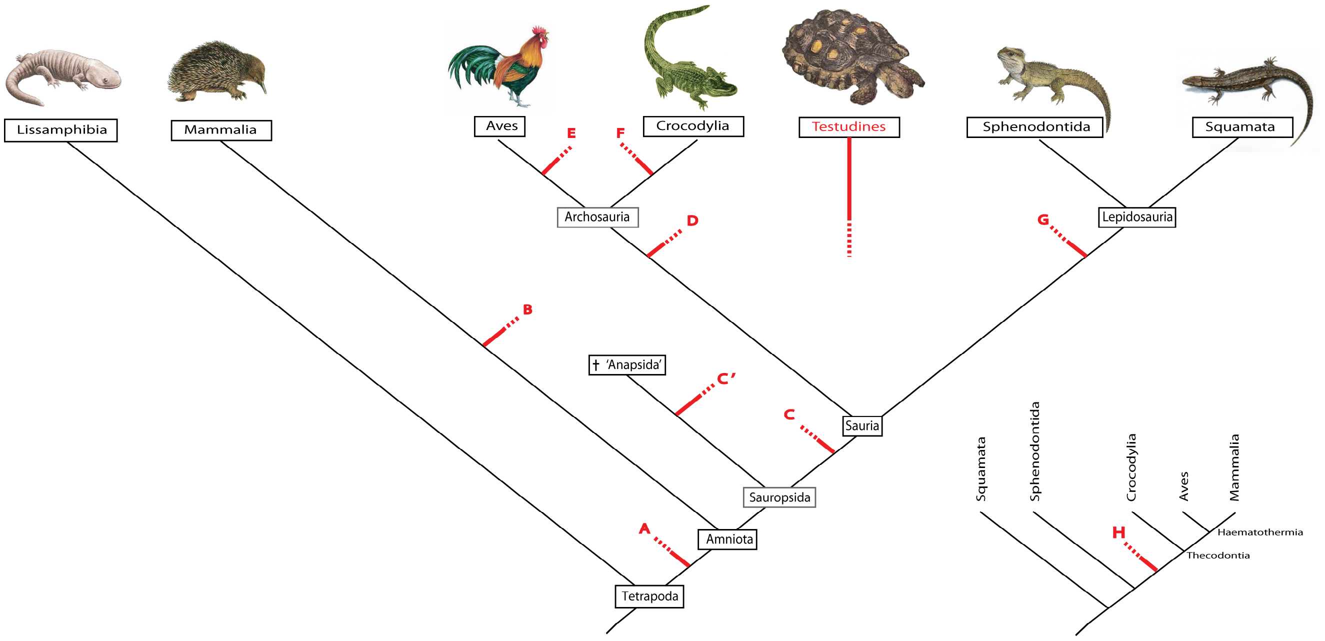

Figure S1 – Alternative turtle positions

All hypotheses for the position of turtles within Tetrapoda summarised as tested in this study (A–H, modified from Figure 1). For references and results each hypothesis see Table S6 and S7. Images modified from different sources and representing used species.

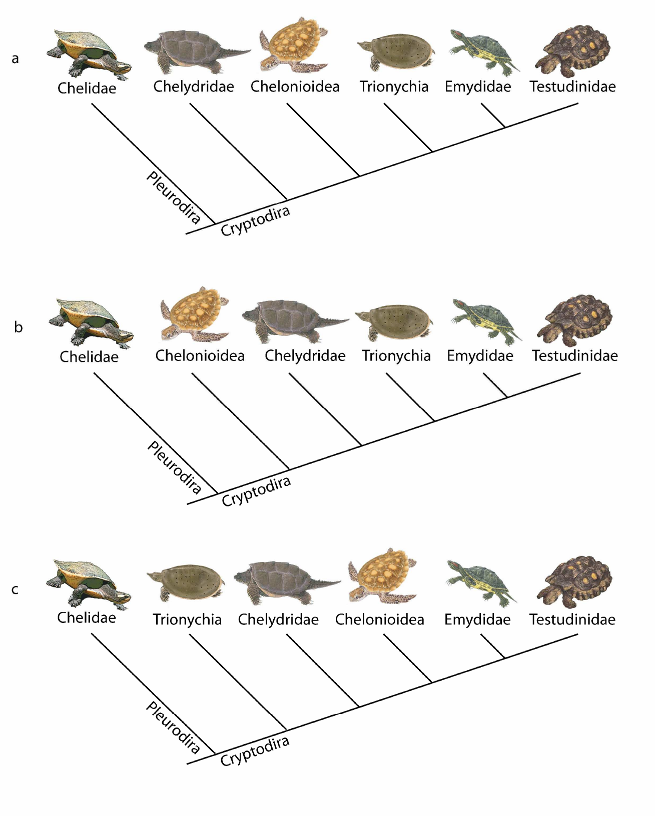

Figure S2 – Alternative cryptodire arrangements

Alternative hypotheses for the relationships of turtle subgroups as tested in this study (modified from Figure 1). a–c show different cryptodire groups as basal taxa. For references and results each tree combination see Table S6 and S7. Images modified from different sources and representing used species.

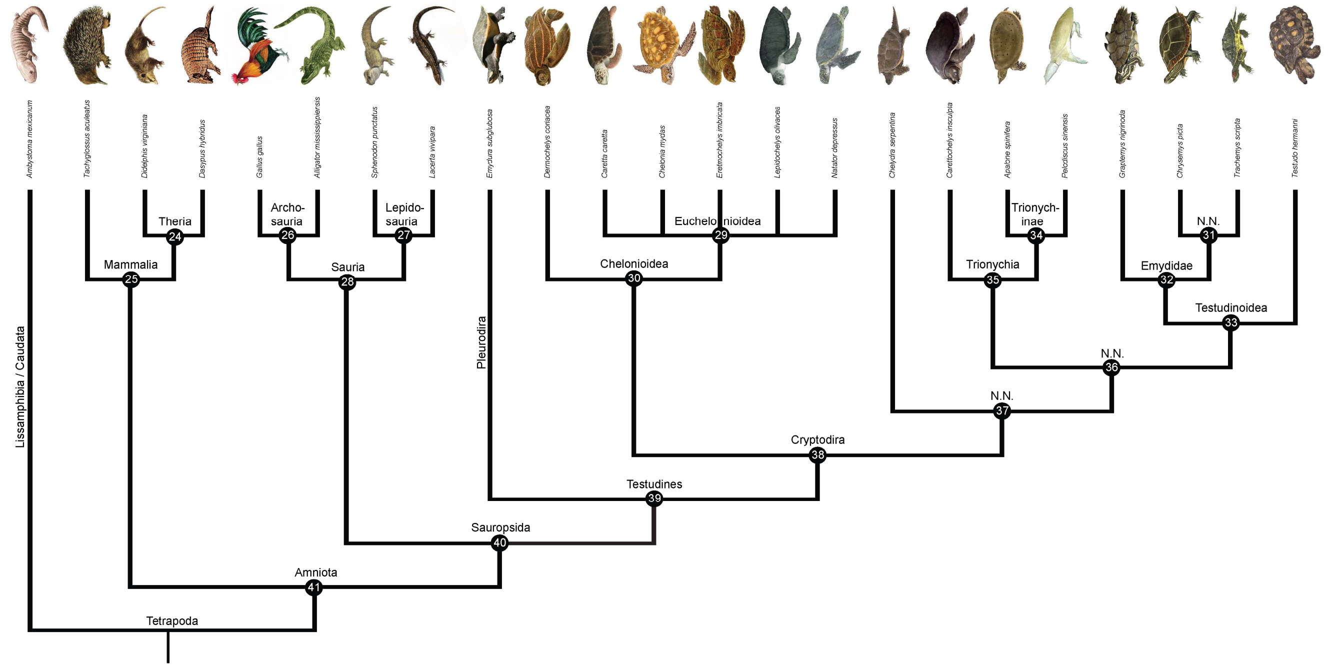

Figure S3 – Best-supported topology

Best-supported topology (modified from Figure 4) including the numbers of nodes as referred in Table S8. Images modified from different sources and representing used species.

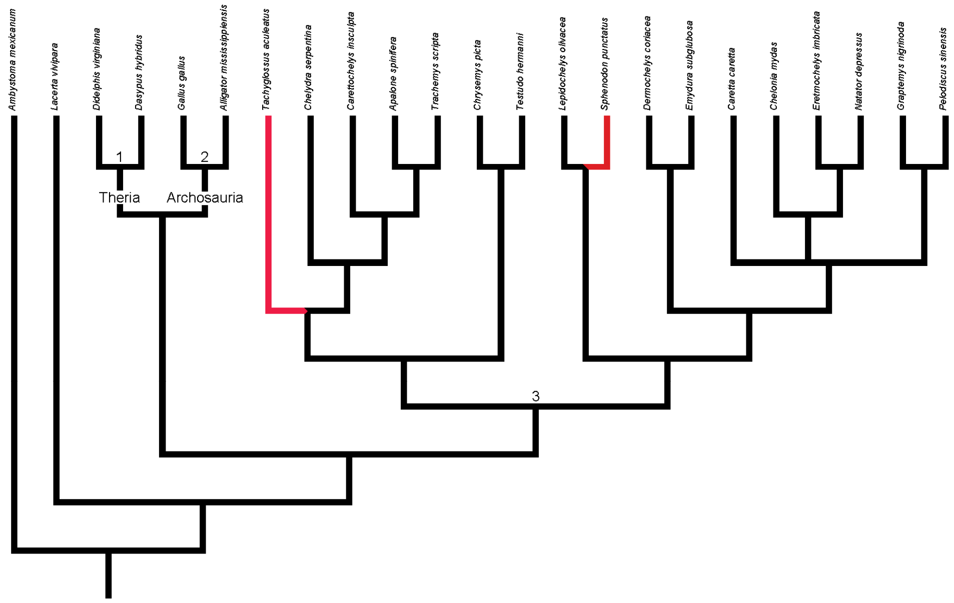

Figure S4 – Phylogenetic reconstruction resulting from an event pair based PAUP* analysis using unordered characters

Strict consensus of two equal parsimonious trees obtained from a parsimony analysis of event pair scores (unweight, **unordered**). Only the labeled nodes reflect the generally accepted arrangements (1, 2). Node 3 indicates turtles as a paraphyletic group including the echidna and tuatara (red). Although there is no consensus about the arrangement of cryptodire subgroups the interrelationships among species in those clades are generally accepted [see 38] – this PAUP* analysis does not reflect any accepted interspecies relationship. This result underlines the problem of using non-independent characters (event pairs) for phylogenetic reconstructions. Tree length = 5887, consistency index (CI) = 0.5466, CI excluding uninformative characters = 0.4491, Homoplasy index (HI) = 0.4534, HI excluding uninformative characters = 0.5509, retention index (RI) = 0.3416, rescaled consistency index (RC) = 0.1867.

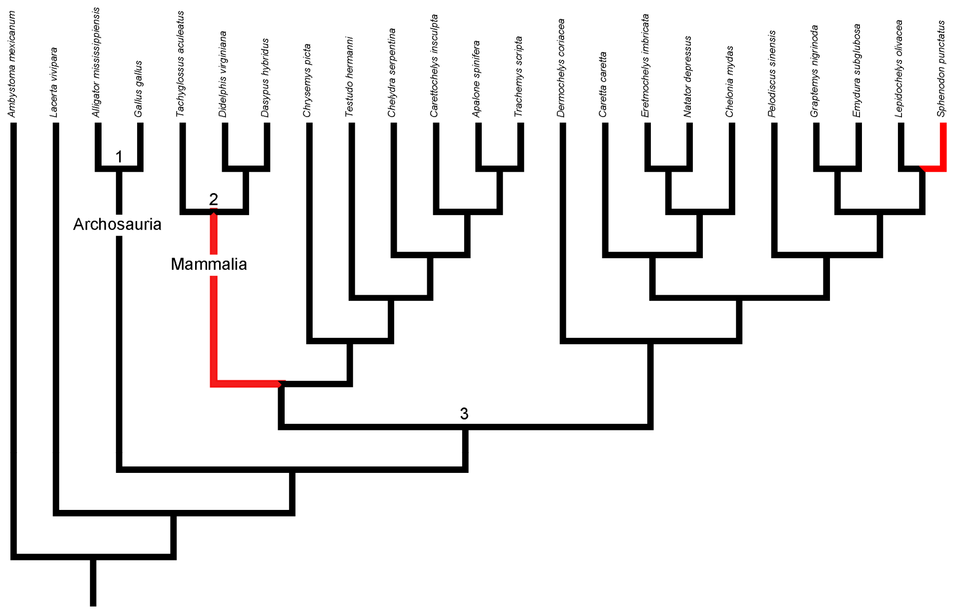

Figure S5 – Phylogenetic reconstruction resulting from an event pair based PAUP* analysis using ordered characters

The parsimonious most tree obtained from a parsimony analysis of event pair scores (unweight, **ordered**). Only the labeled nodes reflect the generally accepted arrangements (1, 2). Node 3 indicates turtles as a paraphyletic group including mammals and tuatara (red). Although there is no consensus about the arrangement of cryptodire subgroups the interrelationships among species in those clades are generally accepted [see 38] – this PAUP* analysis does not reflect any accepted interspecies relationship. This result underlines the problem of using non-independent characters (event pairs) for phylogenetic reconstructions. Tree length = 8056, consistency index (CI) = 0.4683, CI excluding uninformative characters = 0.3855, Homoplasy index (HI) = 0.5317, HI excluding uninformative characters = 0.6145, retention index (RI) = 0.3651, rescaled consistency index (RC) = 0.1710.

References to supplementary information

1. Bordzilovskaya NP, Dettlaff TA, Huhon ST, Malacinski GM: **Developmental–stage series of Axolotl embryos**. In: *Developmental biology of the Axolotl.* Edited by Armstrong JB, Malacinski GM. New York, Oxford: Oxford University Press; 1989: 201–219.
2. Nye HLD, Cameron JA, Chernoff E–AG, Stocum L: **Extending the table of stages of normal development of Axolotl: Limb development**. *Developmental Dynamics* 2003, **226**:555–560.
3. Semon R: **Beobachtungen über die Lebensweise und Fortpflanzung der Monotremen nebst Notizen über ihre Körpertemperatur**. *Denkschriften der Medicinisch–Naturwissenschaftlichen Gesellschaft zu Jena* 1894a, **5**:3–15.
4. Semon R: **Die Embryonalhüllen der Monotremen und Marsupialier**. *Denkschriften der Medicinisch–Naturwissenschaftlichen Gesellschaft zu Jena* 1894b, **5**:19–58.
5. Semon R: **Zur Entwickelungsgeschichte der Monotremen**. *Denkschriften der Medicinisch–Naturwissenschaftlichen Gesellschaft zu Jena* 1894c, **5**:61–74.
6. McCrady E, Jr.: **The embryology of the opossum**. *The American Anatomical Memoirs* 1938, **16**:225.
7. Fernandez M: **Die Entwicklung der Mulita – La embriología de la Mulita (*Tatusia hybrida* Desm.)**. *Revista del Museo de la Plata* 1915, **21**:519.
8. Hamburger V, Hamilton HL: **A series of normal stages in the development of the chick embryo**. *Journal of Morphology* 1951, **88**:49–92.
9. Ferguson MWJ: **Reproductive biology and embryology of the crocodilians**. In: *Biology of the Reptilia Volume 14 – Development A.* Edited by Gans C, Billet F, Maderson PFA. New York: John Wiley & Sons; 1985: 329–491.
10. Voeltzkow A: **Beiträge zur Entwicklungsgeschichte der Reptilien. I. Biologie und Entwicklung der äußeren Körperform von *Crocodilus madagascariensis***. *Abhandlungen der Senckenbergischen Naturforschenden Gesellschaft* 1899, **26**:1–150.
11. Dendy A: **Outlines of the Development of the Tuatara, *Sphenodon (Hatteria) punctatus***. *Quarterly Journal of Microscopical Science* 1899, **s2–42**(165):1–87.
12. Moffat LA: **Embyronic development and aspects of reproductive biology in the tuatara, *Sphenodon punctatus***. In: *Biology of the Reptilia Volume 14 – Development A.* Edited by Gans C, Billet F, Maderson PFA. New York: John Wiley & Sons; 1985: 493–521.
13. Dufaure JP, Hubert J: **Table de développement du lézard vivipara: *Lacerta* (*Zootoca*) *vivipara***. *Archives d'Anatomie Microscopique et de Morphologie Expérimentale* 1961, **50**(3):307–327.
14. Hubert J: **Embryology of the Squamata**. In: *Biology of the Reptilia Volume 15 – Development B.* Edited by Gans C, Billet F, Maderson PFA. New York: John Wiley & Sons; 1985: 1–34.
15. Billett FS, Collins P, Goulding DA, Sutherland J: **The development of *Caretta caretta*, at 25–34°C, in artificial nests**. *Journal of Morphology* 1992, **213**:251–263.
16. Miller JD: **Embryology of marine turtles**. In: *Biology of the Reptilia Volume 14 – Development A.* Edited by Gans C, Billet F, Maderson PFA. New York: John Wiley & Sons; 1985: 269–328.
17. Parker WK: **Report on the development of the green turtle (*Chelone viridis,* Schneid.)**, vol. Zoology 1 (Part 5). Green: London Longmans; 1880.
18. Renous S, Rimblot–Baly F, Fretey J, Pieau C: **Caractéristique du développement embryonnaire de la tortue luth, *Dermochelys coriacea* (Vandelli, 1761)**. *Annales des Sciences Naturelles Zoologie Paris* 1989, **10**:197–229.
19. Crastz F: **Embryological stages of the marine turtle *Lepidochelys olivacea***. *Rev Biol Trop* 1982, **30**:113–120.
20. Yntema CL: **A Series of Stages in the Embrynonic Development of *Chelydra serpentina***. *Journal of Morphology* 1968, **125**(2):219–251.
21. Mahmoud IY, Hess GL, Klicka J: **Normal Embryonic Stages of the Western Painted Turtle, *Chrysemys picta bellii***. *J Morph* 1973, **141**(3):268–280.

22. Greenbaum E: **A standardized series of embryonic stages for the emydid turtle *Trachemys scripta***. *Can J Zool* 2002, **80**:1350–1370.
23. Guyot G, Pieau C, Renous S: **Développement embryonnaire d'une tortue terrestre, la tortue d'Hermann, *Testudo hermanni* Gmelin, 1789**. *Annales des Sciences Naturelles Zoologie Paris* 1994, **15**:115–137.
24. Greenbaum E, Carr JL: **Staging Criteria for Embryos of the Spiny Softshell Turtle, *Apalone spinifera* (Testudines: Trionychidae)**. *Journal of Morphology* 2002, **254**:272–291.
25. Beggs K, Young J, Georges A, West P: **Ageing the eggs and embryos of the pig–nosed turtle, *Carettochelys insculpta* (Chelonia: Carettochelydidae), from northern Australia**. *Canadian Journal of Zoology* 2000, **78**:373–392.
26. Tokita M, Kuratani S: **Normal Embryonic Stages of the Chinese Softshelled Turtle *Pelodiscus sinensis* (Trionychidae)**. *Zoological Science* 2001, **18**:705–715.
27. Werneburg I.: **A standard system to study vertebrate embryos**. PLoS One 2009, in press
28. Jeffery JE, Bininda–Emonds ORP, Coates MI, Richardson MK: **A new technique for identifying sequence heterochrony**. *Systematic Biology* 2005, **54**(2):230–240.
29. Ziermann J: **Evolutionäre Entwicklung larvaler Cranialmuskulatur der Anura und der Einfluss von Sequenzheterochronien.** *PhD–thesis*, Friedrich–Schiller–Universität Jena, Biologisch–Pharmazeutische Fakultät; 2008.
30. Maddison WP, Maddison DR: **Mesquite: a modular system for evolutionary analysis**. Version 2.01; 2007.
31. Swofford DL: **PAUP*. Phylogenetic Analysis Using Parsimony (*and Other Methods)**. Version 4.0b10. Sunderland, Massachusetts: Sinauer Associates; 2003.
32. Homepage of Olaf R. P. Bininda-Emonds, currently: http://www.uni-oldenburg.de/molekularesystematik/
33. Smith KK: **Comparative patterns of craniofacial development in eutherian and metatherian mammals**. *Evolution* 1997, **51**(5):1663–1678.
34. Gaffney ES: **Phylogenetic relationships of the major groups of amniotes**. In: *The Terrestrial Environment and the Origin of Land Vertebrates.* Edited by Panchen A, vol. Special Volume 15. London, New York: Academic Press; 1980.
35. Gaffney ES, McKenna MC: **A Late Permian Captorhinid from Rhodesia**. *American Museum Novitates* 1979, **2688**:1–15.
36. Olson EC: **The family Diadectidae and its bearing on the classification of turtles**. *Fieldiana Geology* 1947, **11**(1):1–53.
37. Laurin M, Reisz RR: **A reevaluation of early amniote phylogeny**. *Zoological Journal of the Linnean Society* 1995, **113**(2):165–223.
38. Osborn HF: **On the primary division of the Reptilia into two sub–classes, Synapsida and Diapsida**. *Science* 1903, **17**(424):275–276.
39. Gaffney ES, Meylan PA: **A phylogeny of turtles**. In: *The Phylogeny and Classification of the Tetrapods Volume 1: Amphibians, Reptiles, Birds.* Edited by Benton MJ, Special Vol. 35A. Oxford: Clarendon Press; 1988: 157–219.
40. Cao Y, Adachi J, Hasegawa M: **Comment on the quartet puzzling method for finding maximum–likelihood tree topologies**. *Molecular Biology and Evolution* 1998, **15**(1):87–89.
41. Rieppel O: **Kontroversen innerhalb der Tetrapoda – die Stellung der Schildkröten (Testudines)**. *Sitzungsberichte der Gesellschaft Naturforschender Freunde zu Berlin* 2004, **43**:201–221.
42. Gaffney ES: **The comparative osteology of the Triassic turtle *Proganochelys***. *Bulletin of the American Museum of Natural History* 1990, **194**:263.
43. Gauthier J, Kluge AG, Rowe T: **Amniote phylogeny and the importance of fossils**. *Cladistics* 1988, **4**:105–209.
44. Gregory WK: **Pareiasaurs versus placodonts as near ancestors to the turtles**. *Bulletin of the American Museum of Natural History* 1946, **86**(Article 6):277–326.
45. Lee MSY: **The origin of the turtle body plan: bridging a famous morphological gap**. *Science* 1993, **261**(5129):1716–1720.

46. Lee MSY: **Correlated progression and the origin of turtles**. *Nature* 1996, **379**(6568):812–815.
47. Lee MSY: **Pareiasaur phylogeny and the origin of turtles**. *Zoological Journal of the Linnean Society* 1997, **120**:197–280.
48. Reisz RR, Laurin M: ***Owenetta* and the Origin of Turtles**. *Nature* 1991, **349**(6307):324–326.
49. Romer AS: **Notes and Comments on Vertebrate Paleontology**. Chicago: The University of Chicago Press; 1968.
50. Williston SW: **The phylogeny and classification of reptiles**. *Journal of Geology* 1917, **25**(5):411–421.
51. de Beer GR: *The Development of the Vertebrate Skull*. Chicago & London: The University of Chicago Press; 1937 (reprint 1985).
52. von Hofsten N: **On the phylogeny of the Reptilia**. *Zool Bidr Uppsala* 1941, **20**:501–521.
53. Hughes S, Zelus D, Mouchiroud D: **Warm–Blooded Isochore Structure in Nile Crocodile and Turtle**. *Mol Biol Evol* 1999, **16**(11):1521–1527.
54. Iwabe N, Hara Y, Kumazawa Y, Shibamoto K, Saito Y, Miyata T, Katoh K: **Sister group relationship of turtles to the bird–crocodilian clade revealed by nuclear DNA–coded proteins**. *Molecular Biology and Evolution* 2004, **22**(4):810–813.
55. Kumazawa Y, Nishida M: **Complete mitochondrial DNA sequences of the green turtle and blue–tailed mole skink: statistical evidence for archosaurian affinity of turtles**. *Molecular Biology and Evolution* 1999, **16**(6):784–792.
56. Matsuda Y, Nishida–Umehara C, Tarui H, Kuroiwa A, Yamada K, Isobe T, Ando J, Fujiwara A, Hirao Y, Nishimura O *et al*: **Highly conserved linkage homology between birds and turtles: bird and turtle chromosomes are precise counterparts of each other**. *Chromosome Research* 2005, **13**(6):601–615.
57. Meyer A, Zardoya R: **Recent advances in the (molecular) phylogeny of vertebrates**. *Annual Review of Ecology, Evolution, and Systematics* 2003, **34**:311–338.
58. Platz JE, Conlon JM: **...and turn back again**. *Nature* 1997, **389**:246.
59. Rest JSR, Ast JC, Austin CC, Waddell PJ, Tibbetts EA, Hay JM, Mindell DP: **Molecular systematics of primary reptilian lineages and the tuatara mitochondrial genome**. *Molecular Phylogenetics and Evolution* 2003, **29**:289–297.
60. Scheyer TM: **Comparative bone histology of the turtle shell (carapace and plastron): implications for turtle systematics, functional morphology and turtle origins**. *PhD–thesis*. Rheinische Friedrich–Wilhelms–Universität Bonn; 2007.
61. Zardoya R, Meyer A: **Complete mitochondrial genome suggests diapsid affinities of turtles**. *Proceedings of the National Academy of Sciences USA* 1998, **95**:14226–14231.
62. Zardoya R, Meyer A: **The evolutionary position of turtles revised**. *Naturwissenschaften* 2001, **88**:193–200.
63. Goodrich ES: **On the classification of the Reptilia**. *Proceedings of the Royal Society of London, Series B, containing papers of a Biological Character* 1916, **89**(615):261–276.
64. Goodrich ES: **Studies on the structure & development of vertebrates**. London: Macmillan and Co.; 1930.
65. Pollock DD, Eisen JA, Doggett NA, Cummings MP: **A case for evolutionary genomics and the comprehensive examination of sequence biodiversity**. *Molecular Biology and Evolution* 2000, **17**(12):1776–1788.
66. Cao Y, Sorenson MD, Kumazawa Y, Mindell DP, Hasegawa M: **Phylogenetic position of turtles among amniotes: evidence from mitochondrial and nuclear genes**. *Gene* 2000, **259**:139–148.
67. Hedges SB, Poling LL: **A molecular phylogeny of reptiles**. *Science* 1999, **283**:998–1001.
68. Mannen H, Li SS–L: **Molecular evidence for a clade of turtles**. *Molecular Phylogenetics and Evolution* 1999, **13**(1):144–148.
69. Baur G: **On the morphology of the vertebrate skull**. *Journal of Morphology* 1889, **3**:471–474.
70. Broom R: **On the classification of the reptiles**. *Bulletin of the American Museum of Natural*

History 1924, **51**:39–65.
71. Hedges SB: **Molecular evidence for the origin of birds**. *Proceedings of the National Academy of Science USA* 1994, **91**:2621–2624.
72. Müller J: **Early loss and multiple return of the lower temporal arcade in diapsid reptiles**. *Naturwissenschaften* 2003, **90**:473–476.
73. Rieppel O: **Studies on skeleton formation in reptiles: Patterns of ossification in the skeleton of *Chelydra serpentina* (Reptilia, Testudines)**. *J Zool Lond* 1993, **231**:487–509.
74. Rieppel O: **Studies on skeleton formation in reptiles: implications for turtle relationships**. *Zoology* 1994/95, **98**:298–308.
75. Rieppel O: **Turtles as diapsid reptiles**. *Zoologica Scripta* 2000, **29**:199–212.
76. Rieppel O, deBraga M: **Turtles as diapsid reptiles**. *Nature* 1996, **384**:453–455.
77. deBraga M, Rieppel O: **Reptile phylogeny and the interrelationships of turtles**. *Zoological Journal of the Linnean Society* 1997, **120**:281–354.
78. Rieppel O, Reisz RR: **The origin and early evolution of turtles**. *Annu Rev Ecol Syst* 1999, **30**:1–22.
79. Gardiner BG: **Tetrapod classification**. *Zoological Journal of the Linnean Society* 1982, **74**:207–232.
80. Gardiner BG: **Haematothermia: warm–blooded amniotes**. *Cladistics* 1993, **9**(4):369–395.
81. Løvtrup S: **On the classification of the taxon Tetrapoda**. *Systematic Zoology* 1985, **34**:463–470.
82. Shaffer HB, Meylan P, McKnight ML: **Tests of turtle phylogeny: Molecular, morphological, and paleontological approaches**. *Systematic Biology* 1997, **46**:234–268.
83. Krenz JG, Naylor GJP, Shaffer BS, Janzen FJ: **Molecular phylogenetics and evolution of turtles**. *Molecular Phylogenetics and Evolution* 2005, **37**:178–191.
84. Jamniczky HA: **Turtle carotid circulation: a character analysis case study**. *Biological Journal of the Linnean Society* 2008, **93**:239–256.
85. Joyce WG: **Phylogenetic relationships of Mesozoic turtles**. *Bulletin of the Peabody Museum of Natural History* 2007, **48**(1):3–102.
86. Rieppel O: **The relationships of turtles within amniotes**. In: *Biology of Turtles.* Edited by Wyneken J, Godfrey MH, Bels V. Boca Raton, London, New York: CRC Press; 2008: 345–353.
